# Associations of Aerobic Fitness and Maximal Muscular Strength With Metabolites in Young Men

**DOI:** 10.1001/jamanetworkopen.2019.8265

**Published:** 2019-08-23

**Authors:** Urho M. Kujala, Jani P. Vaara, Heikki Kainulainen, Tommi Vasankari, Elina Vaara, Heikki Kyröläinen

**Affiliations:** 1 Faculty of Sport and Health Sciences, University of Jyväskylä, Jyväskylä, Finland; 2 Department of Leadership and Military Pedagogy, National Defence University, Helsinki, Finland; 3 The UKK Institute, Centre for Health Promotion Research, Tampere, Finland; 4 JAMK University of Applied Sciences, Jyväskylä, Finland

## Abstract

**Importance:**

High physical fitness is associated with a reduction in risk of cardiovascular diseases and death, but the underlying mechanisms are insufficiently understood.

**Objective:**

To determine how aerobic fitness and muscular strength are associated with serum metabolome measures.

**Design, Setting, and Participants:**

This cross-sectional study included Finnish men receiving military refresher training from May 5, 2015, to November 28, 2015, representing partly overlapping groups of individuals with the lowest vs highest aerobic fitness and the lowest vs highest muscular strength. Data analyses were conducted from January 1, 2018, to May 31, 2019.

**Main Outcomes and Measures:**

The associations of aerobic fitness (determined with maximum oxygen consumption in milliliters per minute per kilogram, measured with maximal cycle ergometer test) and muscular strength (determined with a maximal strength test for lower extremities in kilograms) with 66 metabolome measures from fasting serum samples (nuclear magnetic resonance–based metabolomics) were analyzed.

**Results:**

Participants included 580 Finnish men (mean [SD] age, 26.1 [6.5] years). Including overlap between groups, there were 196 men in the lowest aerobic fitness group and 197 men in the highest aerobic fitness group as well as 196 men in the lowest muscular strength group and 197 men in the highest muscular strength group. Of 66 studied metabolome measures, 48 differed between high vs low aerobic fitness groups, including small very low-density lipoprotein (standardized median difference, −0.67; 95% CI, −0.83 to −0.49), large high-density lipoprotein (standardized median difference, 0.89; 95% CI, 0.69-1.15), total triglyceride levels (standardized median difference, −0.52; 95% CI, −0.65 to −0.34), isoleucine (standardized median difference, −0.37; 95% CI, −0.55 to −0.16), leucine (standardized median difference, −0.55; 95% CI, −0.72 to −0.34), phenylalanine (standardized median difference, −0.54; 95% CI, −0.71 to −0.32), glycerol (standardized median difference, −0.64; 95% CI, −0.81 to −0.48), and glycoprotein (standardized median difference, −0.78; 95% CI, −0.95 to −0.62) concentration, a high unsaturation degree of fatty acids (standardized median difference, 0.59; 95% CI, 0.42-0.81), and apolipoprotein B to apolipoprotein A1 ratio (standardized median difference, −0.88; 95% CI, −1.08 to −0.67). Adding aerobic fitness into the regression model after age, education, smoking, use of alcohol, and dietary factors accounted for more than an additional 5% of variation for 25 metabolome measures (*R*^2^ range, 5.01%-15.90% by measure). With these 2 criteria, maximal muscular strength was not associated with any metabolome measures. Aerobic fitness was associated with high large high-density lipoprotein particle concentration (*R*^2^, 14.97%; 95% CI, 10.65%-20.85%), low apolipoprotein B to apolipoprotein A1 ratio (*R*^2^, 14.49%; 95% CI, 10.58%-19.51%), and low glycoprotein concentration (*R*^2^, 15.90%; 95% CI, 11.22%-21.51%). Aerobic fitness was also associated with low very low-density lipoprotein, triglyceride, isoleucine, leucine, phenylalanine, glycerol, and glycoprotein concentrations and with a high unsaturation degree of fatty acids. Adjusting for recent physical activity influenced the results minimally. Adjusting for body fat percentage showed that some of the associations were mechanistically associated with body fat percentage.

**Conclusions and Relevance:**

This study provides data on the association of high aerobic fitness with underlying oxidative lipid metabolism associated with a reduction in cardiometabolic risk. High maximal muscular strength is not similarly associated with these benefits.

## Key Points

**Question:**

Are aerobic fitness and maximal muscular strength associated with metabolites that are associated with cardiometabolic disease risk?

**Findings:**

In this cross-sectional study of 580 young Finnish men, after adjusting for covariates, aerobic fitness accounted for more than an additional 5% of the variation of 25 serum metabolome measures that are associated with a reduction in cardiometabolic risk. There were fewer beneficial associations of maximal muscular strength with the studied metabolic risk factors.

**Meaning:**

Aerobic fitness was associated with beneficial levels of metabolites associated with reduced vascular and metabolic disease risk.

## Teaser

This cross-sectional study of young Finnish men examines the associations of aerobic fitness and muscular strength with metabolome measures that are associated with cardiometabolic risks.

## Introduction

Aerobic fitness, also called *cardiorespiratory fitness* in clinical medicine, is a measure of the body’s ability to take oxygen from the atmosphere and use it for energy production in the cells. Maximum oxygen consumption (V̇o_2_max), measured in milliliters per minute per kilogram, is a commonly used measure of aerobic fitness. Many factors influence aerobic fitness, including sex, age, genetic factors, body composition, diseases, physical training background, pulmonary and cardiac functions, neural factors, and skeletal muscle properties. Maximal muscular strength is the ability of a muscle or muscle group to generate maximal force. One repetition maximum, measured in kilograms, is a commonly used measure of muscular strength. Aside from the properties of skeletal muscle, maximal muscular strength is mainly determined by muscle mass as well as the number of active motor units and their firing rate. At the muscular level, high aerobic fitness is associated with high capacity for oxidative lipid metabolism, or muscular endurance. Muscular endurance is defined as the muscle’s ability to exert successive submaximal force for a certain time and is also influenced by aerobic fitness.

According to several observational studies,^1,2,3,4,5,6,7,8,9,10^ aerobic fitness, muscular strength, and participation in physical activity measured in different ways are associated with better cardiometabolic health and lower risk of death. According to epidemiological evidence,^1,2,3,7^ aerobic fitness in particular is an indicator of good health and reduced risk of premature death. Aerobic fitness is a stronger predictor of reduced risk of death than physical activity level both in human and in animal studies.^4,11,12,13^ In a 2003 study^14^ of former top-level Finnish male athletes and matched control participants, endurance sports athletes with proven high aerobic capacity had lower occurrence of type 2 diabetes and cardiovascular diseases and reduced risk of death compared with power athletes or control participants. However, genetic pleiotropy may explain part of these associations.^13^

Aerobic fitness is associated with cardiometabolic risk factors.^15,16^ Some studies,^17,18,19^ but not all,^20^ have reported an association of cardiovascular risk factors with muscular strength, but these associations attenuated after adjusting for aerobic fitness.^17,18,21^ In addition to traditional cardiometabolic risk factors measured from the blood, novel metabolomics platforms provide a way to understand the mechanisms associated with the development of diseases. Recently, it has been shown how differing physical activity levels are associated with serum metabolome measures.^22,23,24^

To our knowledge, little research has been conducted on the associations of physical fitness with serum metabolome measures using valid measures of aerobic fitness and muscular strength among humans without diseases or predisease conditions.^25,26^ The skeletal muscle system is the largest and metabolically active organ system in the human body and contributes to the serum metabolome measures associated with aerobic and muscular fitness.^22^ The purpose of this study was to investigate how measured aerobic fitness and maximal muscular strength are associated with serum metabolome among 580 young Finnish men to better understand the mechanisms mediating the association of high physical fitness with low morbidity and mortality.

## Methods

### Participants

**Figure 1.**
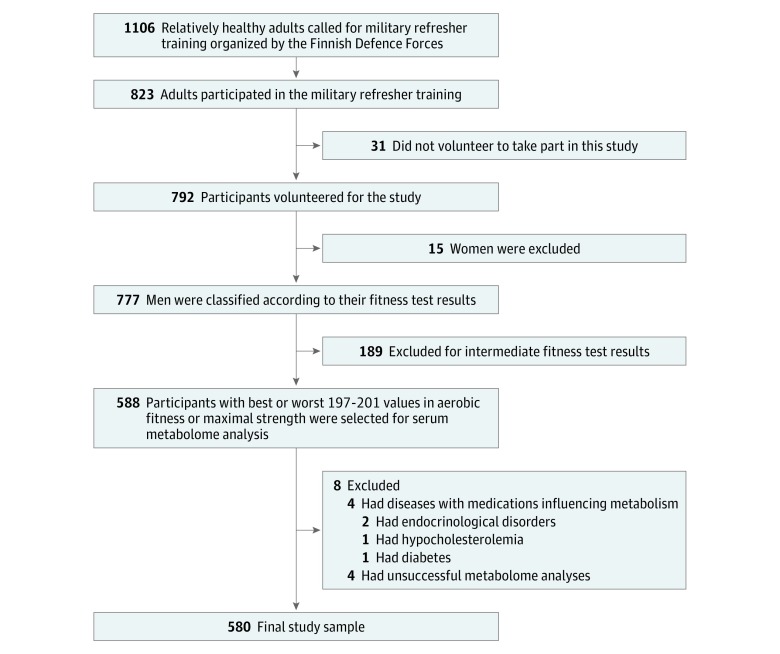
Flowchart of Study Cohort

Study participants were identified from the participants of a previous clinical study of 776 participants in military refresher training from May 5, 2015, to November 28, 2015, during which aerobic fitness and muscular strength were measured and blood samples collected (Figure 1; eAppendix 1 in the Supplement). We identified a group of men with the highest aerobic fitness (approximately 200 participants with the highest fitness test results) and a group with the lowest aerobic fitness (approximately 200 participants with the lowest fitness test results) as well as a corresponding group with the highest muscular strength (approximately 200 participants with the highest strength test results) and a group with the lowest muscular strength (approximately 200 participants with the lowest strength test results). Before fitness tests, all participants underwent exercise eligibility screening, and each included participant was able to exercise to subjective maximum without disease-related symptoms interrupting the test. Individuals with (nonsevere) hypertension, nonoptimal lipid levels, occasional symptoms of asthma or allergy, diabetes, or musculoskeletal symptoms not interfering with maximal exercise were included. Seven participants were taking medication for hypertension, but none of them were taking β-blocker medication. Participants who had diseases or took medications that influenced metabolism or whose metabolome analyses were unsuccessful were excluded. The study protocol was explained in detail to the participants before they provided written informed consent.

The study was approved by the ethics committees of the University of Jyväskylä and the Central Finland Health Care District as well as the Headquarters of the Finnish Defense Forces. Data analyses were conducted from January 1, 2018, to May 31, 2019. This study was reported following the Strengthening the Reporting of Observational Studies in Epidemiology (STROBE) reporting guideline.

### Demographic, Mediating, and Confounding Variables

The demographic and confounding variables assessed using structured questions included age, education, working status, use of alcohol, smoking, dietary habits (including food frequency questions on vegetable, fruit, fish, chicken, and meat consumption), and physical activity (Table 1; eAppendix 2 in the Supplement). Body mass and height were measured on a commercial scale to the closest 0.1 kg and 0.1 cm, respectively. Body mass index was calculated as weight in kilograms divided by height in meters squared, and waist circumference was measured using a cloth tape measure at the level of the iliac crest after exhaling. Body composition was determined after an overnight fast using bioelectrical impedance analysis (InBody 720; InBody) to determine fat mass, body fat percentage, and lean mass. The bioelectrical impedance analysis estimates of body composition have been shown to strongly correlate with the dual-energy radiography absorptiometry method (*r* range, 0.82-0.95).^27^

**Table 1.** Participant Characteristics

Characteristic	Aerobic Fitness			Muscular Strength		
	Low (n = 196)^a^	High (n = 197)^a^	*P* Value^b^	Low (n = 196)^c^	High (n = 197)^c^	*P* Value^b^
Age, y, mean (SD)	26.8 (6.9)	25.0 (5.8)	.007	25.4 (6.4)	27.0 (6.4)	.02
V̇O_2_max, mL/min/kg, mean (SD)	31.8 (3.8)	50.7 (4.2)	<.001	40.2 (7.6)	41.7 (7.9)	.05
Muscular strength, kg, mean (SD)	334 (85)	347 (94)	.15	240 (20)	460 (69)	<.001
Muscular strength/body mass, kg/kg, mean (SD)	3.7 (0.8)^d^	4.8 (1.2)^e^	<.001	3.3 (0.6)^f^	5.4 (1.2)^g^	<.001
Height, cm, mean (SD)	179.8 (6.6)	179.2 (5.8)	.33	178.6 (6.0)	180.5 (6.5)	.003
Body mass, kg, mean (SD)	92.3 (17.9)^d^	73.1 (9.0)^e^	<.001	73.5 (12.7)^f^	88.3 (15.1)^g^	<.001
BMI, mean (SD)	28.5 (4.9)^d^	22.7 (2.3)^e^	<.001	23.0 (3.7)^f^	27.1 (4.2)^g^	<.001
Waist circumference, cm, mean (SD)	96.8 (12.4)	80.3 (5.9)	<.001	83.0 (10.4)	90.9 (11.1)	<.001
Lean mass, kg, mean (SD)	39.0 (5.9)^d^	36.6 (4.4)^e^	<.001	34.2 (4.4)^f^	41.1 (4.7)^g^	<.001
Fat mass, kg, mean (SD)	23.8 (11.6)^h^	9.0 (4.2)^e^	<.001	13.1 (8.3)^f^	16.6 (10.7)^g^	<.001
Body fat, %, mean (SD)	24.8 (8.1)^d^	12.4 (5.6)^e^	<.001	16.9 (7.9)^f^	17.9 (8.0)^g^	.23
Obese (BMI ≥30), No./total No. (%)	71/192 (37.0)	0/194	<.001	10/194 (5.2)	36/196 (18.4)	<.001
Waist circumference >102 cm, No./total No. (%)	67/196 (34.2)	0/197	<.001	8/196 (4.1)	31/197 (15.7)	<.001
Education, No./total No. (%)						
≤Vocational school	116/194 (59.8)	86/196 (43.9)	.002	114/194 (58.8)	95/195 (48.7)	.05
≥Upper secondary school	78/194 (40.2)	110/196 (56.1)		80/194 (41.2)	100/195 (51.3)	
Employment status, No./total No. (%)						
Working	129/194 (66.5)	128/196 (65.3)	.008	116/194 (59.8)	152/196 (77.6)	<.001
Student	44/194 (22.7)	61/196 (31.1)		58/194 (29.9)	40/196 (20.4)	
Unemployed or other	21/194 (10.8)	7/196 (3.6)		20/194 (10.3)	4/196 (2.0)	
Smoking, No./total No. (%)						
Never	79/194 (40.7)	116/196 (59.2)	<.001	97/194 (50.0)	97/196 (49.5)	<.001
Quit	46/194 (23.7)	47/196 (24.0)		31/194 (16.0)	61/196 (31.1)	
Regular	69/194 (35.6)	33/196 (16.8)		66/194 (34.0)	38/196 (19.4)	
Use of alcohol, No./total No. (%)						
Never	11/194 (5.7)	31/196 (15.8)	.29	14/194 (7.2)	14/196 (7.1)	.65
≤1-2 Times/wk	143/194 (73.7)	138/196 (70.4)		142/194 (73.2)	150/196 (76.5)	
≥3 Times/wk	40/194 (20.6)	27/196 (13.8)		38/194 (19.6)	32/196 (16.3)	
≥6 Drinks on 1 occasion, ≥1 time/wk	52/194 (26.8)	48/196 (24.5)	.64	54/194 (27.8)	44/196 (22.4)	.24
Diet ≥3 d/wk, No./total No. (%)						
Fresh vegetables	102/194 (52.6)	125/196 (63.8)	.03	99/194 (51.0)	138/196 (70.4)	<.001
Cooked vegetables	41/194 (21.1)	53/196 (27.0)	.19	42/194 (21.6)	65/196 (33.2)	.01
Fruits or berries	56/194 (28.9)	102/196 (52.0)	<.001	53/194 (27.3)	94/196 (48.0)	<.001
Chicken	47/194 (24.2)	64/196 (32.7)	.07	36/194 (18.6)	69/196 (35.2)	<.001
Fish	12/194 (6.2)	12/196 (5.9)	.10	12/194 (6.2)	13/196 (6.6)	.10
Meat	137/194 (70.6)	150/196 (76.5)	.21	134/194 (69.1)	143/196 (73.0)	.44
Processed	35/194 (18.0)	44/196 (22.4)	.31	40/194 (20.6)	25/196 (12.8)	.04
Total amount of physical activity/wk, h, mean (SD)						
Light aerobic	4.3 (9.2)^g^	6.8 (13.0)	.03	4.2 (8.5)^g^	5.6 (11.4)	.14
Moderate aerobic	2.4 (5.4)^f^	3.1 (5.6)	.20	2.6 (6.1)^f^	2.7 (6.1)	.90
Vigorous aerobic	0.9 (1.9)^f^	2.6 (2.8)	<.001	1.1 (1.8)^f^	1.8 (2.3)	<.001
Strength training	1.1 (3.0)^f^	2.0 (2.6)	.001	0.7 (1.5)^f^	2.4 (2.9)	<.001
Sum of moderate to vigorous aerobic training	3.3 (6.2)^f^	5.7 (6.5)	<.001	3.7 (6.3)^f^	4.5 (6.5)	.20
Work-related loading, No./total No. (%)						
Sedentary work	53/192 (27.6)	41/193 (21.2)	.42	52/193 (26.9)	53/188 (28.2)	.88
Walking and other light activity	28/192 (14.6)	35/193 (18.1)		35/193 (18.1)	29/188 (15.4)	
Walking and lifting material	64/192 (33.3)	63/193 (32.6)		56/193 (29.0)	59/188 (31.4)	
Heavy manual work	47/192 (24.5)	54/193 (28.0)		50/193 (25.9)	47/188 (25.0)	
Blood pressure, mm Hg, mean (SD)						
Systolic	127.2 (11.7)	121.8 (11.4)	<.001	119.9 (10.5)	126.5 (12.5)	<.001
Diastolic	78.0 (8.8)	71.7 (8.3)	<.001	73.9 (8.7)	74.6 (10.3)	.47

Abbreviations: BMI, body mass index (calculated as weight in kilograms divided by height in meters squared); V̇O_2_max, maximum oxygen consumption.

^a^ Ranges: low, 15.5-36.6 mL/min/kg; high, 45.4-67.1 mL/min/kg.

^b^ *P* values for the difference between high fitness vs low fitness groups for categorical variables by χ^2^ tests and for continuous variables by permutation median test.

^c^ Ranges: low, 115-279 kg; high, 382-738 kg.

^d^ Data were missing for 4 participants.

^e^ Data were missing for 3 participants.

^f^ Data were missing for 2 participants.

^g^ Data were missing for 1 participant.

^h^ Data were missing for 5 participants.

### Assessment of Physical Fitness

Maximal aerobic fitness, as measured by V̇o_2_max, was determined using an indirect graded cycle ergometer (Ergoline 800S, Ergoselect 100K, and Ergoselect 200K; Ergoline) test until exhaustion. A progressive protocol was used that started at a power output of 50 W and increased 25 W every 2 minutes until exhaustion. Heart rate was continuously recorded during the test using Polar Vantage NV or Polar S610, S710, or S810 heart rate monitors (Polar). Predicted V̇o_2_max was estimated from heart rate and maximal power (W) using Fitware fitness testing software (Fitware) using the equation V̇o_2_max = 12 × 35 × Wattsmax/kg + 3.5, in which Wattsmax indicates the maximal power and kg refers to the unit of body mass. This protocol is accurate (SD, 3%) and reliable to estimate V̇O_2_max values in healthy men, with the reported intraclass correlation being high (*r* range, 0.82-0.94).^28^

The maximal isometric leg extension was measured using a dynamometer as a test of maximal strength.^29^ The repeatability has been reported to be high in maximal isometric strength tests (*r*, 0.98; coefficient of variation, 4.1%).^30^ Additional details are presented in eAppendix 1 in the Supplement.

### Serum Metabolome Measures

Participants’ circulating metabolomes, including lipids, lipoproteins, and metabolites, were assessed using a high-throughput proton nuclear magnetic resonance spectroscopy metabolomics platform.^31,32^ Collectively, the 153 metabolic traits measured by the platform represent a broad molecular signature of systemic metabolism.^31,32^ The platform quantifies various measures of lipoprotein metabolism, certain lipids, ketone bodies, and amino acids, as well as glycolysis and gluconeogenesis-related metabolites in absolute concentration units. Based on our 2013 study^22^ on physical activity and metabolomics, 66 metabolites or their ratios were selected for this study (eTable 2 in the Supplement).

### Statistical Analysis

The primary aim of our study was to examine whether V̇o_2_max per kilogram of body mass and maximal muscular strength without adjusting for body mass or body composition are associated with metabolome. First, we analyzed the unadjusted results of metabolome measures between high vs low aerobic fitness and high vs low muscular strength groups. Second, all of the participants were included in linear regression analyses in which we investigated separately how aerobic fitness and muscular strength (independent continuous variables) were associated with different metabolome measures (dependent continuous variables). The models were first adjusted for age (continuous variable), and education level, smoking, use of alcohol, and indicators of dietary factors (dichotomous variables) (eAppendix 2 in the Supplement). Next, we investigated how adjusting for concomitants of fitness, such as reported physical activity habits (aerobic activities and strength training volumes; continuous variables), and body fat percentage (continuous variable) affected the associations.

As it may be physiologically more relevant to express muscular strength per mass, a comparison of how the results differed when calculated using maximal strength vs maximal strength per body mass was also reported. Maximal strength per body mass was expected to correlate more strongly with maximal aerobic fitness. Finally, we also included aerobic fitness and muscular strength in a regression model to determine which had a stronger correlation with the variation in the metabolome measures.

*P* values were 2-tailed, calculated using χ^2^ tests or permutation median tests, and were not corrected for multiple testing. Our previous estimation of the multiple test correction can be used for the interpretation of statistical significance in all the 66 studied metabolome measures. We used principal component analysis to determine the minimum number of orthogonal linear components from a similar full metabolomics measures panel that explained 99% of the observed variance in a large data set. The minimum number of orthogonal linear components was analyzed, and the highest number observed in the previously studied cohort^22^ was 26 components, which we used as a consistent conservative estimate in all multiple testing interpretations using Bonferroni method. Bonferroni-corrected *P* value of .002 or less was set as the level for statistical significance, which corresponds to a *P* value of less than .05.

The data were analyzed with R statistical software version 3.4.1 (R Project for Statistical Computing) and SPSS statistical software version 25.0 (IBM). Pretreatment of the variables included imputation of the minimum values in instances in which the level of the specific metabolome measure was too small to detect (eTable 2 in the Supplement). Descriptive statistics, such as frequencies, means, and SDs, as well as medians and nonparametric bias-corrected and accelerated bootstrapped 95% CIs for the variance of medians, were calculated. Natural log transformations were used for all of the regression analyses for metabolome measures that were not normally distributed (eTable 2 in the Supplement). Linear regression analyses were conducted to define *R*^2^ as a measure of variance accounted for, as well as change in *R*^2^ after the addition of the variable of interest. For the change in coefficients of determination, bias-corrected and accelerated 95% CIs were computed with *boot* and *boot.ci* from the library *boot* in R (Table 2).

**Table 2.** Variation in Selected Metabolome Measures Accounted for by the Covariates and Physical Fitness

Metabolome Measure	%				
	Model 1 *R*^2^^a^	Model 2 *R*^2^^b^^,^^c^	Model 2−Model 1 *R*^2^ Difference (95% CI)^d^	Model 3 *R*^2^^c^^,^^e^	Model 3−Model 1 *R*^2^ Difference (95% CI)
Lipoprotein particle concentration					
Large VLDL	5.86	12.13 (−)	6.28 (3.27-11.02)	5.91 (+)	0.06 (0-0.69)
Medium VLDL	8.36	17.14 (−)	8.78 (5.08-13.40)	8.83 (+)	0.47 (0-2.16)
Small VLDL	13.27	22.27 (−)	9.00 (5.47-13.46)	14.01 (+)	0.74 (0.01-2.51)
Very small VLDL	18.24	24.37 (−)	6.13 (3.49-9.54)	19.13 (+)	0.89 (0.02-2.62)
Very large HDL	5.53	13.97 (+)	8.43 (5.19-13.11)	5.76 (−)	0.23 (0-1.47)
Large HDL	8.21	23.18 (+)	14.97 (10.65-20.85)	8.93 (−)	0.72 (0.02-2.78)
Lipoprotein particle size					
VLDL diameter	4.68	10.94 (−)	6.27 (3.11-10.48)	4.81 (+)	0.13 (0-1.28)
HDL diameter	8.90	23.35 (+)	14.45 (10.21-20.00)	9.40 (−)	0.50 (0-2.22)
APOs					
APOB	14.65	22.91 (−)	8.26 (5.10-12.38)	15.55 (+)	0.90 (0.03-2.95)
APOB:APOA1 ratio	15.20	29.69 (−)	14.49 (10.58-19.51)	16.47 (+)	1.27 (0.12-3.75)
TGs					
Total TG	10.34	20.07 (−)	9.73 (5.95-14.55)	10.86 (+)	0.52 (0-2.31)
VLDL TG	8.60	18.17 (−)	9.57 (5.82-14.22)	9.02 (+)	0.42 (0-2.09)
IDL TG	15.38	21.60 (−)	6.22 (3.21-10.17)	15.72 (+)	0.34 (0-1.65)
Cholesterol					
VLDL	13.60	21.30 (−)	7.70 (4.49-11.85)	14.40 (+)	0.80 (0.01-2.77)
HDL	5.72	17.19 (+)	11.47 (7.81-16.90)	6.61 (−)	0.89 (0.04-3.20)
HDL subfraction 2	7.07	19.34 (+)	12.27 (8.34-17.80)	7.96 (−)	0.89 (0.06-3.12)
FAs					
Unsaturation degree	9.05	17.27 (+)	8.22 (4.64-13.07)	9.17 (+)	0.13 (0-1.26)
ω-6 FA ratio	8.15	16.64 (+)	8.49 (4.78-13.83)	8.66 (−)	0.51 (0-2.38)
Saturated FA	13.78	18.79 (−)	5.01 (2.32-8.82)	14.14 (+)	0.36 (0-1.65)
Monounsaturated FA	13.56	19.97 (−)	6.41 (3.38-10.55)	13.78 (+)	0.22 (0-1.34)
Metabolic substrates, amino acids, and other					
Glycerol	4.03	20.19 (−)	16.17 (10.81-22.79)	4.52 (−)	0.50 (0-2.17)
Isoleucine (BCAA)	0.90	7.85 (−)	6.95 (3.77-11.22)	1.42 (+)	0.52 (0-2.53)
Leucine (BCAA)	2.04	8.91 (−)	6.88 (3.58-10.79)	2.82 (+)	0.78 (0.01-3.25)
Phenylalanine	4.06	10.52 (−)	6.46 (3.10-11.21)	6.08 (+)	2.02 (0.36-4.91)
Glycoproteins	6.21	22.11 (−)	15.90 (11.22-21.51)	6.38 (+)	0.17 (0-1.54)

Abbreviations: APO, apolipoprotein; BCAA, branched-chain amino acid; FA, fatty acid; HDL, high-density lipoprotein; IDL, intermediate-density lipoprotein; TG, triglyceride; VLDL, very low-density lipoprotein.

^a^ Model 1 *R*^2^ from linear regression analysis shows how much age, education, smoking, use of alcohol, and dietary factors (consumption of vegetables, fruits, fish, chicken, and meat) account for the variation in each of the metabolome measures.

^b^ Model 2 *R*^2^ shows how much age, education, smoking, use of alcohol, dietary factors (consumption of vegetables, fruits, fish, chicken, and meat), and aerobic fitness account for the variation in each of the metabolome measures.

^c^ + and – indicate the direction of the association of the fitness variable with each metabolome measure.

^d^ Statistically significant additional *R*^2^ value with *P* ≤ .002.

^e^ Model 3 *R*^2^ shows how much age, education, smoking, use of alcohol, dietary factors (consumption of vegetables, fruits, fish, chicken, and meat), and muscular strength account for the variation in each of the metabolome measures.

## Results

From 588 individuals selected for having the best or worst values in aerobic fitness or maximal strength, we excluded 4 participants having a disease or using medication that influenced metabolism and 4 participants owing to unsuccessful metabolome analyses (Figure 1). As there was overlap between the aerobic fitness and muscular strength groups (eTable 1 in the Supplement), the total cohort included 580 adult white Finnish men (mean [SD] age, 26.1 [6.5] years). Our final groups included 196 participants with low aerobic fitness (V̇o_2_max range, 15.5-36.6 mL/min/kg), 197 participants with high aerobic fitness (V̇o_2_max range, 45.4-67.1 mL/min/kg), 196 participants with low muscular strength (maximal muscular strength range, 115-279 kg), and 197 participants with high muscular strength (maximal muscular strength range, 382-738 kg) (Table 1). Compared with participants with high aerobic fitness, participants with low aerobic fitness, had a higher mean (SD) body mass index (28.5 [4.9] vs 22.7 [2.3]) and a larger mean (SD) waist circumference (96.8 [12.4] cm vs 80.3 [5.9] cm). Compared with participants with low muscular strength, participants with high muscular strength had a higher mean (SD) body mass index (27.1 [4.2] vs 23.0 [3.7]) and a larger mean (SD) waist circumference (90.9 [11.1] cm vs 83.0 [10.4] cm). Participants with high aerobic fitness had the highest rate of upper secondary school educations or higher (110 participants [56.1%]). Participants with high muscular strength had the highest rate of working at the time of study participation (152 participants [77.6%]). Both types of fitness were associated with more mean (SD) moderate to vigorous aerobic training (high aerobic fitness, 5.7 (6.2) h/wk; low aerobic fitness, 3.3 [6.2] h/wk; high muscular strength, 4.5 [6.5] h/wk; low muscular strength, 3.7 [6.3] h/wk), more mean (SD) strength training (high aerobic fitness, 2.0 (2.6) h/wk; low aerobic fitness, 1.1 [3.0] h/wk; high muscular strength, 2.4 [2.9] h/wk; low muscular strength, 0.7 [1.5] h/wk), and lower incidence of regular smoking (high aerobic fitness, 33 participants [16.8%]; low aerobic fitness, 69 participants [35.6%]; high muscular strength, 38 participants [19.4%]; low muscular strength, 66 participants [34.0%]) (Table 1).

**Figure 2.**
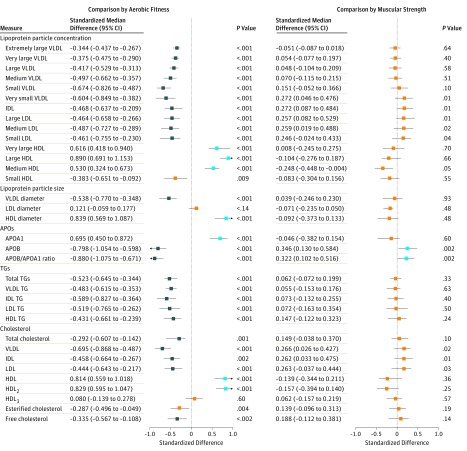
Serum Metabolome Measures in Individuals With High Fitness Compared With Individuals With Low Fitness According to Aerobic Fitness and Muscular Strength The points and lines indicate the differences in individuals with high fitness compared with individuals with low fitness in medians and their bootstrapped 95% CIs scaled in SDs of the pooled data of all 580 participants. Blue indicates serum levels that were statistically significantly higher among individuals with high fitness compared with those with low fitness; navy, serum levels that were statistically significantly lower among individuals with high fitness compared with those with low fitness; and orange, differences were not statistically significant. APO indicates apolipoprotein; HDL, high-density lipoprotein; HDL_2_, HDL subfraction 2; HDL_3_, HDL subfraction 3; IDL, intermediate-density lipoprotein; LDL, low-density lipoprotein; TG, triglyceride; VLDL, very low-density lipoprotein.

**Figure 3.**
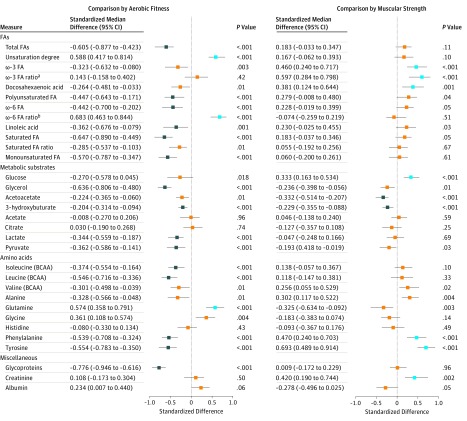
Serum Metabolome Measures in Individuals With High Fitness Compared With Individuals With Low Fitness According to Aerobic Fitness and Muscular Strength The points and lines indicate the differences in individuals with high fitness compared with individuals with low fitness in medians and their bootstrapped 95% CIs scaled in SDs of the pooled data of all 580 participants. Blue indicates serum levels that were statistically significantly higher among individuals with high fitness compared with those with low fitness; navy, serum levels that were statistically significantly lower among individuals with high fitness compared with those with low fitness; and orange, differences were not statistically significant. BCAA indicates branched-chain amino acid; FA, fatty acid. ^a^ω-3 FA ratio is the ratio of ω-3 FAs to total FAs. ^b^ω-6 FA ratio is the ratio of ω-6 FAs to total FAs.

Nonadjusted differences in serum metabolome measures by aerobic fitness and muscular strength groups are shown in Figure 2 and Figure 3 and in eTable 3 and eTable 4 in the Supplement. There were many differences between the participants with high aerobic fitness vs low aerobic fitness but fewer differences between those with high muscular strength vs low muscular strength. The largest median differences between the high aerobic fitness vs low aerobic fitness groups relative to pooled SDs were found for large high-density lipoprotein (HDL) particles (median standardized difference, 0.89; 95% CI, 0.69-1.15; *P* < .001), the ratio of apolipoproteins (APOs) APOB to APOA1 (median standardized difference, −0.88; 95% CI, −1.08 to −0.67; *P* < .001), and glycoproteins (median standardized difference, −0.78; 95% CI, −0.95 to −0.62; *P* < .001). According to age-adjusted regression analysis among the 580 participants, the findings were in accordance with the group comparisons (eTable 5 and eTable 6 in the Supplement).

The associations of fitness characteristics with metabolome measures were similar in most cases after adjusting for age, education level, smoking, use of alcohol, and dietary factors (Table 2; eTable 7 and eTable 8 in the Supplement). Twenty-five of the 66 studied metabolome measures differed between high vs low aerobic fitness groups (Figure 2 and Figure 3; eTable 3 and eTable 4 in the Supplement), and among the 580 men, adding aerobic fitness into the regression model after age, education, smoking, use of alcohol, and dietary factors accounted for more than an additional 5% of their variation (*R*^2^ range, 5.01%-15.90%) (Table 2; eTable 7 in the Supplement). Within these 2 criteria, maximal muscular strength was not associated with any metabolome measures. After adjusting for covariates, aerobic fitness was associated with high large HDL particle concentration (*R*^2^, 14.97%; 95% CI, 10.65-20.85), with low APOB/APOA1 ratio (*R*^2^, 14.49%; 95% CI, 10.58-19.51), and with low glycoprotein concentration (*R*^2^, 15.90%; 95% CI, 11.22-21.51). The eFigure in the Supplement shows a correlation heat map of fitness characteristics with metabolome measures, of which variation was accounted for by at least 5% by aerobic fitness after adjusting for the main covariates. All of the variables are linked to fat metabolism.

Most associations of physical fitness with metabolome measures remained statistically significant with only small changes in the explanation rates after adjusting for physical activity (eTable 9 and eTable 10 in the Supplement). After adjusting for age and body fat percentage, aerobic fitness was statistically significantly associated with 19 of the included metabolome measures, including higher levels of large HDL particles (*R*^2^, 4.15%; 95% CI, 1.75%-7.33%) and APOA1 (*R*^2^, 3.25%; 95% CI, 1.05%-6.39%); lower levels of medium very low-density lipoprotein (*R*^2^, 1.36%; 95% CI, 0.25%-3.76%), very low-density lipoprotein triglycerides (*R*^2^, 1.72%; 95% CI, 0.33%-4.12%), and total triglycerides (*R*^2^, 1.49%; 95% CI, 0.24%-3.77%); unsaturation degree of fatty acids (FAs) (*R*^2^, 2.62%; 95% CI, 0.98%-4.84%); and lower levels of glycerol (*R*^2^, 3.17%; 95% CI, 0.88%-8.18%), acetoacetate (*R*^2^, 3.76%; 95% CI, 1.26%-7.54%), 3-hydroxybuturate (*R*^2^, 2.63%; 95% CI, 0.56%-6.25%), and glycoproteins (*R*^2^, 2.76%; 95% CI, 0.80%-5.88%) (eTable 11 in the Supplement). After adjusting for age and body fat percentage, muscular strength was associated with 8 of the measures, including high levels of ω-3 FAs (*R*^2^, 1.58%; 95% CI, 0.26%-3.58%), phenylalanine (*R*^2^, 1.72%; 95% CI, 0.32%-4.00%), tyrosine (*R*^2^, 4.74%; 95% CI, 2.08%-8.18%), and creatinine (*R*^2^, 5.90%; 95% CI, 2.39%-10.41%) and low levels of glycerol (*R*^2^, 1.74%; 95% CI, 0.29%-4.02%), acetoacetate (*R*^2^, 3.55%; 95% CI, 1.14%-7.10%), and 3-hydroxybuturate (*R*^2^, 3.53%; 95% CI, 0.96%-6.58%) (eTable 12 in the Supplement). A summary of the changes in the explanation rates when adding different covariates to the models is shown in eTable 13 in the Supplement.

The correlation between maximal aerobic fitness and maximal muscular strength was stronger when muscular strength was expressed per body mass (*r* = 0.43; 95% CI, 0.37-0.49; *P* < .001) compared with when muscular strength was expressed as an absolute value (*r* = 0.09; 95% CI, 0.01-0.15; *P* = .04). The unfavorable associations of metabolome measures with muscle strength were usually attenuated when we used muscular strength expressed per body mass (eTable 14 in the Supplement). After adjusting for age, body fat percentage, aerobic fitness, and maximal muscular strength per body mass, we found that the associations with aerobic fitness largely persisted, but after these adjustments, muscular strength was only associated with high creatinine concentration (eTable 15 in the Supplement).

## Discussion

This study found many associations of high aerobic fitness with metabolome measures, indicating reduced cardiometabolic risk in relatively healthy young men, while maximal muscular strength had fewer such associations with metabolome measures. Many of the associations were mechanistically associated with body fat percentage. To our knowledge, there are no previous similar studies with similarly targeted metabolomics platforms.

The associations of lower very low-density lipoprotein, intermediate-density lipoprotein, and low-density lipoprotein particle concentrations as well as lower triglyceride levels with high aerobic fitness are similar to the findings in 2 previous studies^22,33^ of physically active individuals that suggested decreased risk of coronary heart disease. Body fat percentage explained or mediated much of these associations. After adjusting for body fat percentage, aerobic fitness explained an additional 4.2% of the variation in the content of large HDL particles. Although drugs that increase HDL cholesterol levels are not effective in reducing cardiovascular disease risk, high HDL cholesterol levels were associated with low risk of coronary heart disease in a review by Natarajan et al,^34^ and several studies^35,36,37^ have shown that HDL cholesterol levels increase with exercise. In particular, the functionally beneficial larger HDL particle concentration is increased in physically active individuals.^22^ Our findings are in agreement with studies by Kujala et al^22^ and Blazek et al^37^ that reported that high aerobic fitness or high physical activity was associated with the improved function of HDL particles. In addition to previously known mechanisms,^37^ the function of HDL particles may be associated with high oxygen uptake and associated muscle metabolism.^22,38^ Further research is warranted to explain these mechanisms, as there is evidence that suggests HDL modulates skeletal muscle cellular respiration.^38^

Aerobic fitness was associated with lower levels of most types of FA concentrations, including total and saturated FA, monounsaturated FA, and ω-6 FA, unlike maximum strength, which was associated with higher docosahexaenoic acid and ω-3 FA concentrations. Interestingly, after adjusting for body fat percentage, aerobic fitness correlated more closely with the degree of unsaturation than with any other FA parameter. High aerobic fitness was associated with a free FA profile, which is a characteristic of reduced cardiovascular event risk.^39^ However, substituting ω-6, ω-3, or total polyunsaturated FAs for saturated FAs in the diet is not very efficient in reducing cardiovascular disease risk.^40,41,42,43^ Our results suggest that fitness characteristics themselves at least partly define the favorable serum FA profile but also that some FAs may influence fitness characteristics.^44^

Lower serum lactate and pyruvate concentrations in the participants with high aerobic fitness compared with the participants with low aerobic fitness are most likely owing to several interacting factors. First, more oxidative muscle fibers can exist more frequently in skeletal muscles of aerobically fit people owing to heredity^45^ or training,^46^ thus using more lactate for energy production than glycolytic fibers.^47^ Skeletal muscle mitochondria use lactate for oxidation more efficiently than they use pyruvate, suggesting that glucose is first largely metabolized to lactate, secreted out of muscle cells, and only then transported back to the same or other cells.^48^ This supports the idea that type IIB fibers produce lactate that is consumed by type I and IIA fibers for energy production and that this cycle is more efficient in people who are aerobically fit. Second, in participants who were aerobically fit, skeletal muscle glycogenesis for restoring glycogen stores at rest may have been more efficient than in those who were less fit. Third, hepatic gluconeogenesis from lactate is likely higher in people who are aerobically fit than in those who are less fit.^49^

Interestingly, high aerobic fitness and high muscle strength were associated with lower levels of glycerol and ketone bodies acetoacetate and 3-hydroxybutyrate, suggesting increased fat catabolism. A 2015 study^50^ found that lipolysis-derived serum glycerol levels at rest can be lower in aerobically trained individuals than in untrained individuals,^50^ similar to this study, but 2 older studies^51,52^ reported no observed difference. Differences in the results can be explained by variations in glycerol kinetics, as glycerol appearance and disappearance rates are considerably higher in people who have undergone endurance training compared with those who have not.^52^ Lower glycerol levels at rest may be owing to enhanced hepatic gluconeogenesis from glycerol in individuals who are physically fit. Ketone bodies are mainly produced in the liver from FAs released from adipose tissue and used for energy production (eg, in the skeletal muscles, heart, and brain). Skeletal muscle accounts for the highest amount of the use of ketone bodies.^53^ In individuals who were aerobically fit, lower serum ketone body concentrations may be associated with increased activity of ketolytic enzymes in the skeletal muscles, as seen in rodents.^54^ In people with high muscle strength, the lower ketone body levels may be explained by higher muscle mass capable of using ketone bodies for energy.

Increased serum levels of branched-chain amino acids have been shown to predict the occurrence of type 2 diabetes^55^ and are associated with physical inactivity and unfavorable lipid metabolism.^22^ This is most likely because branched-chain amino acids degradation mechanistically connects to tricarboxylic acid cycle, intramyocellular lipid storage, and oxidation, thus allowing more efficient mitochondrial energy production from lipids as well as providing better metabolic health.^56^

In our study and in a 2013 study^57^ examining the association of aerobic fitness with metabolic profiles, serum phenylalanine concentration was negatively associated with high aerobic fitness, supporting the finding of Würtz et al,^39^ in which a high serum phenylalanine level was associated with the increased risk of cardiovascular events. Acute phenylalanine supplementation combined with exercise is associated with increased glucagon concentration and increased whole body fat oxidation.^58^ This implies that lower phenylalanine concentration in individuals who are aerobically fit may be due to more efficient removal of phenylalanine from the bloodstream to enhance fat oxidation. To our knowledge, the details of the mechanism for this effect of phenylalanine remain to be shown. Glycoprotein acetyls, in particular α1-acid glycoprotein, are indicators of glycosylation modification of secreted inflammatory proteins and have been reported to predict mortality.^59,60^ In this study, the creatinine levels were higher among those with high muscular strength and high skeletal muscle mass.

### Limitations

This study used carefully validated methods to measure the fitness characteristics, but the maximal aerobic fitness test method was an indirect way to estimate V̇o_2_max. Furthermore, the participants were relatively healthy white Finnish men, which led to little confounding due to diseases. It is unclear how our results could be generalized to individuals from other races, women, and older people with noncommunicable diseases. Estrogens, aging, development of diseases, and development of nonalcoholic fatty liver disease may influence the associations of physical fitness with metabolomics.^61,62^

## Conclusions

This study shows confirmatory and novel associations of aerobic fitness with vascular and metabolic disease risk factors already present in young, relatively healthy men. Many of the mechanisms may be associated with skeletal muscle metabolism, body fat content, and metabolism. Although the most beneficial associations were mostly seen for aerobic fitness, muscular endurance training also increases aerobic fitness. In addition, muscular training is an important component in maintaining or improving function among patients with long-term diseases and in the later years of life.^63^ Further interventional research is needed on whether the observed associations are more genetically determined or whether improving fitness by physical training has beneficial influences on health.

## Notes

Supplement. **eAppendix 1.** Study Plan **eAppendix 2.** Questionnaire Items on Covariates Used in the Regression Analyses **eFigure.** Heat Map of Correlations Between Fitness Characteristics and Metabolome Measures **eTable 1.** Number of Participants in the Overlapping High Fitness vs Low Aerobic Fitness and High Muscular Strength vs Low Muscular Strength Groups **eTable 2.** List of Analyzed Metabolome Measures, Their Abbreviations, and Number of Imputed Values **eTable 3.** Serum Metabolome Measures by Aerobic Fitness and Muscular Strength Groups in SI Units **eTable 4.** Differences in the Medians of Serum Metabolome Measures Between Aerobic Fitness Groups and Between the Muscular Strength Groups Relative to 1 SD of Each Variable **eTable 5.** Linear Regression Analysis of the Age-Adjusted Association of Aerobic Fitness With Serum Metabolome Measures **eTable 6.** Linear Regression Analysis of the Age-Adjusted Association of Maximal Muscular Strength With Serum Metabolome Measures **eTable 7.** Linear Regression Analysis of the Age-, Education-, Smoking-, Use of Alcohol-, and Diet-Adjusted Association of Aerobic Fitness With Serum Metabolome Measures **eTable 8.** Linear Regression Analysis of the Age-, Education-, Smoking-, Use of Alcohol-, and Diet-Adjusted Association of Maximal Muscular Strength With Serum Metabolome Measures **eTable 9.** Linear Regression Analysis of the Age-Adjusted and Physical Activity–Adjusted Association of Aerobic Fitness With Serum Metabolome Measures **eTable 10.** Linear Regression Analysis of the Age-Adjusted and Physical Activity–Adjusted Association of Maximal Muscular Strength With Serum Metabolome Measures **eTable 11.** Linear Regression Analysis of the Age-Adjusted and Body Fat Percentage–Adjusted Association of Aerobic Fitness With Serum Metabolome Measures **eTable 12.** Linear Regression Analysis of the Age-Adjusted and Body Fat Percentage–Adjusted Association of Maximal Muscular Strength With Serum Metabolome Measures **eTable 13.** Summary of the Δ*R*^2^ Values From Regression Models Indicating the Additional Variation Accounted for by Each Fitness Variable **eTable 14.** Additional Explanation Rate for Absolute and Relative-to-Body-Weight Maximal Muscular Strength After Adjusting for Age and After Adjusting for Age and Body Fat Percentage **eTable 15.** Regression Models Including Age, Body Fat Percentage, Aerobic Fitness, and Relative Muscular Strength
