# Supplementary material for: Associations of Aerobic Fitness and Maximal Muscular Strength With Metabolites in Young Men
Source: JAMA Netw Open. 2019 Aug 23;2(8):e198265. doi: 10.1001/jamanetworkopen.2019.8265 (PMC6714035; doi:10.1001/jamanetworkopen.2019.8265)
Supplement: Supplement. — eAppendix 1. Study Plan eAppendix 2. Questionnaire Items on Covariates Used in the Regression Analyses eFigure. Heat Map of Correlations Between Fitness Characteristics and Metabolome Measures eTable 1. Number of Participants in the Overlapping High Fitness vs Low Aerobic Fitness and High Muscular Strength vs Low Muscular Strength Groups eTable 2. List of Analyzed Metabolome Measures, Their Abbreviations, and Number of Imputed Values eTable 3. Serum Metabolome Measures by Aerobic Fitness and Muscular Strength Groups in SI Units eTable 4. Differences in the Medians of Serum Metabolome Measures Between Aerobic Fitness Groups and Between the Muscular Strength Groups Relative to 1 SD of Each Variable eTable 5. Linear Regression Analysis of the Age-Adjusted Association of Aerobic Fitness With Serum Metabolome Measures eTable 6. Linear Regression Analysis of the Age-Adjusted Association of Maximal Muscular Strength With Serum Metabolome Measures eTable 7. Linear Regression Analysis of the Age-, Education-, Smoking-, Use of Alcohol-, and Diet-Adjusted Association of Aerobic Fitness With Serum Metabolome Measures eTable 8. Linear Regression Analysis of the Age-, Education-, Smoking-, Use of Alcohol-, and Diet-Adjusted Association of Maximal Muscular Strength With Serum Metabolome Measures eTable 9. Linear Regression Analysis of the Age-Adjusted and Physical Activity–Adjusted Association of Aerobic Fitness With Serum Metabolome Measures eTable 10. Linear Regression Analysis of the Age-Adjusted and Physical Activity–Adjusted Association of Maximal Muscular Strength With Serum Metabolome Measures eTable 11. Linear Regression Analysis of the Age-Adjusted and Body Fat Percentage–Adjusted Association of Aerobic Fitness With Serum Metabolome Measures eTable 12. Linear Regression Analysis of the Age-Adjusted and Body Fat Percentage–Adjusted Association of Maximal Muscular Strength With Serum Metabolome Measures eTable 13. Summary of the ΔR2 Values From Regression Models Indicating [file jamanetwopen-2-e198265-s001.pdf]

Supplementary Online Content

Kujala UM, Vaara JP, Kainulainen H, Vasankari T, Vaara E, Kyröläinen H. Associations of aerobic fitness and maximal muscular strength with metabolites in young men. *JAMA Netw Open*. 2019;2(8):e198265. doi:10.1001/jamanetworkopen.2019.8265

eAppendix 1. Study Plan

eAppendix 2. Questionnaire Items on Covariates Used in the Regression Analyses

eFigure. Heat Map of Correlations Between Fitness Characteristics and Metabolome Measures

eTable 1. Number of Participants in the Overlapping High Fitness vs Low Aerobic Fitness and High Muscular Strength vs Low Muscular Strength Groups

eTable 2. List of Analysed Metabolome Measures, Their Abbreviations, and Number of Imputed Values

eTable 3. Serum Metabolome Measures by Aerobic Fitness and Muscular Strength Groups in SI Units

eTable 4. Differences in the Medians of Serum Metabolome Measures Between Aerobic Fitness Groups and Between the Muscular Strength Groups Relative to 1 SD of Each Variable

eTable 5. Linear Regression Analysis of the Age-Adjusted Association of Aerobic Fitness With Serum Metabolome Measures

eTable 6. Linear Regression Analysis of the Age-Adjusted Association of Maximal Muscular Strength With Serum Metabolome Measures

eTable 7. Linear Regression Analysis of the Age-, Education-, Smoking-, Use of Alcohol-, and Diet-Adjusted Association of Aerobic Fitness With Serum Metabolome Measures

eTable 8. Linear Regression Analysis of the Age-, Education-, Smoking-, Use of Alcohol-, and Diet-Adjusted Association of Maximal Muscular Strength With Serum Metabolome Measures

eTable 9. Linear Regression Analysis of the Age-Adjusted and Physical Activity–Adjusted Association of Aerobic Fitness With Serum Metabolome Measures

eTable 10. Linear Regression Analysis of the Age-Adjusted and Physical Activity–Adjusted Association of Maximal Muscular Strength With Serum Metabolome Measures

eTable 11. Linear Regression Analysis of the Age-Adjusted And Body Fat Percentage–Adjusted Association of Aerobic Fitness With Serum Metabolome Measures

eTable 12. Linear Regression Analysis of the Age-Adjusted And Body Fat Percentage–Adjusted Association of Maximal Muscular Strength With Serum Metabolome Measures

eTable 13. Summary of the ΔR^2 Values From Regression Models Indicating the Additional Variation Accounted for by Each Fitness Variable

eTable 14. Additional Explanation Rate for Absolute and Relative-to-Body-Weight Maximal Muscular Strength After Adjusting for Age and After Adjusting for Age and Body Fat Percentage

eTable 15. Regression Models Including Age, Body Fat Percentage, Aerobic Fitness, and Relative Muscular Strength

This supplementary material has been provided by the authors to give readers additional information about their work.

© 2019 Kujala UM et al. *JAMA Network Open.*

eAppendix 1. Study Plan

Associations of Aerobic fitness and muscular strength with metabolomics in young adult men: a cross-sectional study

Introduction and general study aim
High aerobic fitness (often called as cardiorespiratory fitness in clinical medicine) is a strong biological predictor of reduced risk of vascular and metabolic diseases and reduced risk of death.[1-7] The majority of studies showing these associations have been conducted in elderly populations among whom early stages of disease may have had strengthened the associations due to reverse causality. The aim of our study is to investigate how measured aerobic fitness (calculated maximal oxygen uptake, $ml \times min^{-1} \times kg^{-1}$) and maximal muscular strength (one repetition maximum, kilograms) are associated with metabolome measures among young adult Finnish men. In addition to cardiac and pulmonary health many underlying biological mechanisms including the properties of the skeletal muscles contribute to these fitness characteristics. An important link between physical activity, aerobic fitness, properties of skeletal muscle, and serum metabolome is that both inherited aerobic fitness and physical activity are associated with the increased expression of oxidative phosphorylation and fat metabolism pathways in skeletal muscle.[8-10]

The skeletal muscle is the largest metabolically active organ in the human body and contributes to the aerobic and muscular fitness related serum metabolome.[10] Multi-dimensional metabolome measures may be related to aerobic muscle metabolism that is associated with aerobic fitness and contributes to the mechanisms explaining the associations between aerobic fitness and beneficial cardio-metabolic risk factor profiles.

We hypothesize that our study provides confirmatory data and new insights into how high aerobic fitness compared to low aerobic fitness is associated with oxidative lipid metabolism indicative of reduced cardio-metabolic risk. Our hypothesis includes that high maximal muscular strength is not similarly associated with these benefits as aerobic fitness. Muscular endurance is more strongly associated with aerobic fitness than explosive maximal muscular strength.

Participants and methods
Military service is compulsory for young healthy men in Finland. Approximately 70-75% of men serves in the military. After their military service, the men return to their normal civilian lives. Some may occasionally be called up to military refresher training organized by the Finnish Defence Forces and lasting 2-5 days. The call up for military refresher training is organised by the Finnish Defence Forces. Information regarding the study plan was sent to prospective participants 5 months before the study was conducted. The study was carried out over 7 different sessions during 2015. The study protocol was explained in detail to the participants before they provided written consent. The study was approved by the ethics committees of the University of Jyväskylä and the Central Finland Health Care District, as well as the Headquarters of the Finnish Defence Forces. Briefly, 1106 men and women were called up and 823 agreed to participate in the military refresher training (a response rate of 74%). The most typical reasons for nonparticipation to the military refresher training were related to personal factors, such as work-, study-, or health-related issues. Among those men who participated in the military refresher training 32 refused to take part in the study (a response rate of 96%). In addition, 15 women participated in the study but are not included in these analyses. The study sample was compared with corresponding cohorts of 20 to 30 years old Finnish men in the national register data (Statistics Finland) from 2014 for education and place of residence. Based on these analyses, the current study sample is not fully nationally representative geographically since Northern and Southern Finland were slightly over-represented. In addition, the proportion of those participants who had studied for 13 years or more was slightly over-represented. Taking into account previous limitations, the present study sample can be considered to represent a young adult Finnish man.

From the participants of a structured clinical study of 776 military refresher training subjects, we will identify a group of men with high aerobic fitness (approximately 200 participants with the best results) and a group with low aerobic fitness (approximately 200 with lowest aerobic fitness), and correspondingly a group with highest muscular strength (approximately 200 participants) and with lowest muscular strength (approximately 200 participants). The examinations include responding to a questionnaire on physical activity and different health habits, diseases and medications, maximal aerobic cycle ergometer test for calculation of maximal oxygen uptake, maximal lower limb strength test, and fasting blood samples for metabolomics analysis.

Assessment of physical fitness: Aerobic fitness (VO_{2max}) is determined using a previously validated[11] indirect graded cycle ergometer (Ergoline 800S, Ergoselect 100K, Ergoselect 200K, Ergoline, Bitz, Germany) test until exhaustion. A progressive protocol is used, which starts at a power output of 50 W and is increased 25 W every 2 min until exhaustion. The heart rate (HR) is continuously recorded during the test (Polar Vantage NV or S610, S710, or S810, Polar, Kempele, Finland). The predicted VO_{2max} was estimated from the HR and maximal power (W) (Fitware, Mikkeli, Finland) using the following equation: VO_{2max} ($ml \times min^{-1} \times kg^{-1}$) = $12.35 \times P_{max}/kg + 3.5$, where P_{max} is the maximal power and kg is the body mass in kg. Maximal isometric lower limb strength is measured with leg extension using a dynamometer (regarded as tests for maximal strength).[12] The knee angle is set to 107° with a goniometer, and the hands

are placed on a handle grip. After a warm-up, three maximum trials are performed using a 30-s recovery period. The best performance of a 3 is included in the analysis. Each participant is advised to produce maximal force as fast as possible and to maintain it for three seconds. The participants are verbally encouraged by the test personnel during the maximal efforts.

Body composition is determined using bioelectrical impedance analysis (BIA) (InBody 720, InBody, Seoul, South Korea) after an overnight fast, to determine fat mass (FM), body fat percentage, and lean mass. Body mass and height are measured to the closest 0.1 kg and 0.1 cm, respectively, on a commercial scale. Body mass index (BMI) is calculated, and waist circumference is measured using a cloth tape measure at the level of the iliac crest after exhaling.

Serum metabolome: A profile of the participants' serum metabolome including circulating lipids, lipoproteins, and metabolites is compiled using a high-throughput NMR metabolomics platform.[13,14] Collectively, the 153 metabolic traits measured by the platform represent a broad molecular signature of systemic metabolism.[13,14] The platform provides the simultaneous quantification of lipoprotein lipids and subclasses, FAs and FA compositions, ketone bodies, amino acids, as well as glycolysis and gluconeogenesis-related metabolites in absolute concentration units. This platform has been applied to various large-scale epidemiological and genetic studies.[10,15] The detailed protocol, including information on quality control, has been published elsewhere.[13,16] For this study analysis, we have selected 66 metabolome measures on the basis of our previous study on physical activity and metabolomics.[10] For a complete list of analysed metabolites and their abbreviations, see **eTable 2** in this **Supplement**.

More specific research questions and analysis strategy

On the basis of our previous research on physical activity, fitness, and muscle metabolism,[10] we are particularly interested in how aerobic fitness and explosive muscular strength are associated with lipoprotein particle concentrations, particle sizes and their apolipoproteins and cholesterol, serum fatty acid concentrations, unsaturation degree of fatty acids, glycerol, 3-hydroxybuturate, lactate, pyruvate, acetoacetate, branched-chain amino acids, phenylalanine, tyrosine, glycoproteins, and creatinine.

For the interpretation of statistical significance in our analyses of all the 66 studied metabolome measures our previous estimation of the multiple test correction can be used. The minimum number of orthogonal linear components (PCA) from the similar full metabolomics measures panel that explained 99% of the observed variance in a large dataset was analyzed and the highest number observed in the previously studied cohort[10] was 26 components and we use that as a consistent conservative estimate in all multiple testing interpretations using Bonferroni's method. Bonferroni's corrected P value ≤0.002 corresponds to a statistical significance level of <0.05.

First, in the relatively healthy young male adult study group, we will analyze and visualize the unadjusted results of metabolome measures between high *vs* low aerobic fitness and high *vs* low muscular strength groups. Then we will adjust the results for education, smoking, use of alcohol, and indicators of dietary factors such as the consumption of fruits, vegetables, and meat (chicken, red meat, and fish separately). We will carry out additional analyses to assess how adjustment for such concomitants of fitness as reported physical activity habits (aerobic activities and strength training) and with body fat percentage influence the associations. It is particularly relevant to understand how aerobic fitness is associated with body fat percentage as aerobic fitness and training are associated with reductions in high-risk body fat,[17,18] and changes in body composition influence the measured fitness characteristics when the fitness tests include carrying body mass or the fitness characteristics are expressed per body mass.

Our primary aim is to elucidate whether maximal oxygen uptake in terms of $ml \times kg^{-1} \times min^{-1}$and maximal muscular strength without adjustments for body mass or body composition is associated with metabolome. However, as it may be physiologically relevant to express body strength per body mass, we also report how the results differ when calculated per maximal strength *vs* maximal strength per body mass. Maximal strength per body mass is expected to correlate more strongly with aerobic fitness than the absolute non-proportional strength value.

© 2019 Kujala UM et al. *JAMA Network Open.*

References to study plan

1. Myers J, Prakash M, Froelicher V, Do D, Partington S, Atwood JE. Exercise capacity and mortality among men referred for exercise testing. *N Engl J Med.* 2002;346(11):793–801.
2. Kujala UM, Marti P, Kaprio J, Hernelahti M, Tikkanen H, Sarna S. Occurrence of chronic disease in former top-level athletes. Predominance of benefits, risks or selection effects? *Sports Med.* 2003;33(8):553–561.
3. Kodama S, Saito K, Tanaka S, et al. Cardiorespiratory fitness as a quantitative predictor of all-cause mortality and cardiovascular events in healthy men and women: a meta-analysis. *JAMA.* 2009;301(19):2024–2035.
4. Lee DC, Sui X, Ortega FB, et al. Comparisons of leisure-time physical activity and cardiorespiratory fitness as predictors of all-cause mortality in men and women. *Br J Sports Med.* 2011;45(6):504–510.
5. Karvinen S, Waller K, Silvennoinen M, et al. Physical activity in adulthood: genes and mortality. *Sci Rep.* 2015;5:18259.
6. Ross R, Blair SN, Arena R, et al. Importance of assessing cardiorespiratory fitness in clinical practice: A case for fitness as a clinical vital sign. A scientific statement from the American Heart Association. *Circulation.* 2016;134(24): e653–e699.
7. Kujala UM. Is physical activity a cause of longevity? It's not as straightforward as some would believe. A critical analysis. *Br J Sports Med.* 2018;52(14):914–918.
8. Kivelä R, Silvennoinen M, Lehti M, et al. Gene expression centroids that link with low intrinsic aerobic exercise capacity and complex disease risk. *FASEB J*. 2010;24(11):4565–4574.
9. Leskinen T, Rinnankoski-Tuikka R, Rintala M, et al. Differences in muscle and adipose tissue gene expression and cardio-metabolic risk factors in the members of physical activity discordant twin pairs. *PLoS One.* 2010;5(9):e12609.
10. Kujala UM, Mäkinen V-P, Heinonen I, et al. Long-term leisure-time physical activity and serum metabolome. *Circulation.* 2013;127(3):340–348.
11. Santtila M, Häkkinen K, Pihlainen K, Kyröläinen H. Comparison between direct and predicted maximal oxygen uptake measurement during cycling. *Mil Med.* 2013;178(2):234-238.
12. Häkkinen K, Häkkinen A. Neuromuscular adaptations during intensive strength training in middle-aged and elderly males and females. *Electromyogr Clin Neurophysiol.* 1995;35(3):137–147.
13. Soininen P, Kangas AJ, Wurtz P, et al. High-throughput serum NMR metabonomics for cost-effective holistic studies on systemic metabolism. *Analyst.* 2009;134(9):1781–1785.
14. Soininen P, Kangas AJ, Wurtz P, Suna T, Ala-Korpela M. Quantitative serum nuclear magnetic resonance metabolomics in cardiovascular epidemiology and genetics. *Circ Cardiovasc Genet.* 2015;8(1):192–206.
15. Kettunen J, Tukiainen T, Sarin AP, et al. Genome-wide association study identifies multiple loci influencing human serum metabolite levels. *Nat Genet.* 2012;44(3):269–276.
16. Inouye M, Kettunen J, Soininen P, et al. Metabonomic, transcriptomic, and genomic variation of a population cohort. *Mol Syst Biol.* 2010;6:441.
17. Ohkawara K, Tanaka S, Miyachi M, Ishikawa-Takata K, Tabata I. A dose-response relation between aerobic exercise and visceral fat reduction: systematic review of clinical trials. *Int J Obes.* 2007;31(12):1786–1797.
18. Leskinen T, Sipilä S, Alen M, et al. Leisure time physical activity and high-risk fat: A longitudinal population-based twin study. *Int J Obes.* 2009;33(11):1211–1218.

© 2019 Kujala UM et al. *JAMA Network Open.*

eAppendix 2. Questionnaire Items on Covariates Used in the Regression Analyses

Education

3. What is your highest level of education?

a Comprehensive school (primary school classes 1 to 9)
b Vocational upper secondary school
c Upper secondary school (high school)
d Vocational college diploma
e Degree from university of applied sciences (polytechnic)
f Degree from university (bachelor, master, doctoral or higher)

- ➢ *Dicotomised for regression analysis: education/socioeconomic status (upper secondary school or higher, c-f vs. vocational school or lower, a-b)*

Dietary habits

46 How often during the past week have you eaten chicken?

a Not at all
b On 1-2 days
c On 3-5 days
d On 6–7 days

- ➢ *Chicken consumption dichotomised for regression analysis: eat chicken at least on 3 days a week vs. less often.*

47 How often during the past week have you eaten fish?

a Not at all
b On 1-2 days
c On 3-5 days
d On 6–7 days

- ➢ *Fish consumption dichotomised for regression analysis: eat fish at least on 3 days a week vs. less often.*

48 How often during the past week have you eaten meat?

a Not at all
b On 1-2 days
c On 3-5 days
d On 6–7 days

49 How often during the past week have you eaten processed meat (for example, sausages)?

a Not at all
b On 1-2 days
c On 3-5 days
d On 6–7 days

- ➢ *Dicotomised for regression analysis: eat meat or processed meat at least on 3 days a week vs. less often.*

51. How often during the past week have you eaten fresh vegetables?

a Not at all
b On 1-2 days
c On 3-5 days
d On 6–7 days

52. How often during the past week have you eaten cooked vegetables?

a Not at all
b On 1-2 days
c On 3-5 days
d On 6–7 days

53. How often during the past week have you eaten fruits or berries?

a Not at all
b On 1-2 days
c On 3-5 days
d On 6–7 days

- ➢ *Fruit or vegetable consumption dichotomised for regression analysis: any of the three above at least on 3 days a week vs. less than on 3 days a week in all the three above.*

© 2019 Kujala UM et al. *JAMA Network Open.*

Alcohol
63. How often do you use alcohol? Also take into account those occasions when you use alcohol only in small doses such as one bottle of beer or one glass of wine.
a Daily
b 5-6 times per week
c 3-4 times per week
d 1-2 times per week
e Twice per month
f About once per month
g About once per two months
h 3-4 times per year or less
I I do not use alcohol

- *Three-class reclassified alcohol use for regression analysis: at least 3 times per week, 1-2 times per week, and less than once per week.*

Smoking
64. What are your smoking habits like?
a I have never smoked regularly
b I quit smoking more than 6 months ago
c I quit smoking less than 6 months ago
d I smoke regularly

- *Three-class reclassification for regression analysis: current smokers, quitters,and never smoked regularly.*

Physical activity
In the following question, consider all regular physical activity in your work, commuting and leisure-time.

16. What is your total weekly physical activity volume? Think about the past year (12 months). Take into account all weekly regular physical activity that lasts at least 10 minutes at a time. Circle all of the alternatives corresponding your situation (2-5), and mark on empty rows the volume of each type of your activity you participate in (days per week, hours, and minutes altogether per week).
If you do not participate in physical activity regularly on a weekly basis, choose alternative 1 and leave the other alternatives empty.

1 No regular physical activity weekly
2 Light endurance type of physical activity
(= no sweating or breathlessness, for example, light walking)
On____ days per week, altogether ____ hours ____ minutes per week
3 Brisk moderate intensity endurance type of physical activity
(= some sweating and breathlessness, for example, brisk walking)
On____ days per week, altogether ____ hours ____ minutes per week
4 Vigorous endurance type of physical activity
(= much sweating and breathlessness, for example, jogging or running)
On____ days per week, altogether ____ hours ____ minutes per week
5 Muscle strength training (= for example, circuit training or other type of gym training during which exercise manoeuvre for each muscle group is repeated at least 8-12 times)
On____ days per week, altogether ____ hours ____ minutes per week

- *Physical activity (aerobic moderate to vigorous physical activity (min/week) and strength training (min/week)) were used in the models as continuous variables.*

© 2019 Kujala UM et al. *JAMA Network Open.*

eFigure. Heat Map of Correlations Between Fitness Characteristics and Metabolome Measures[a]

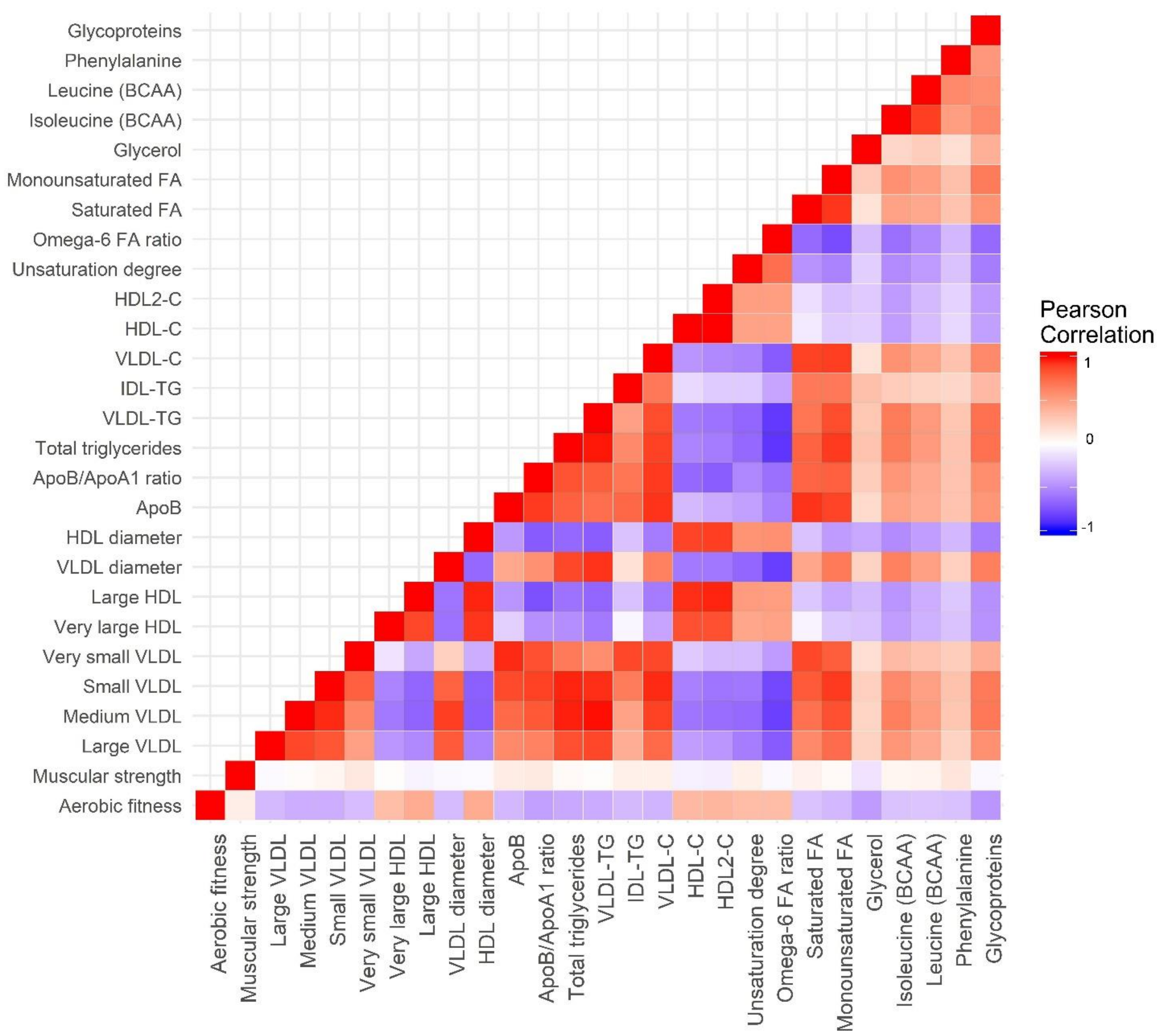

[a]All metabolome measures which variation was accounted for by at least 5% by aerobic fitness variable after adjustment for the main covariates (age, education, smoking, use of alcohol, and diet (fruits and vegetables, meat, fish, and chicken)) are included.

© 2019 Kujala UM et al. *JAMA Network Open.*

eTable 1. Number of Participants in the Overlapping High Fitness vs Low Aerobic Fitness and High Muscular Strength vs Low Muscular Strength Groups[a]

	Aerobic fitness			
**Muscular strength**	**High**	**Intermediate**	**Low**	**Total**
High	55	90	52	197
Intermediate	98	-	89	187
Low	44	97	55	196
Total	197	187	196	580

[a]Total number of participants is 580

© 2019 Kujala UM et al. *JAMA Network Open.*

eTable 2. List of Analysed Metabolome Measures, Their Abbreviations, and Number of Imputed Values

**Name of variable used in tables**	**Explanation**	**Number of imputed values (%)[b]**
**Lipoprotein particle concentration**		
Extremely large VLDL	Concentration of chylomicrons and extremely large VLDL particles	122 (21.0)[c]
Very large VLDL[a]	Concentration of very large VLDL particles	140 (24.1)[c]
Large VLDL[a]	Concentration of large VLDL particles	78 (13.4)[c]
Medium VLDL[a]	Concentration of medium VLDL particles	2 (0.3)
Small VLDL[a]	Concentration of small VLDL particles	0
Very small VLDL	Concentration of very small VLDL particles	1 (0.2)
IDL	Concentration of IDL particles	0
Large LDL	Concentration of large LDL particles	1 (0.2)
Medium LDL	Concentration of medium LDL particles	2 (0.2)
Small LDL	Concentration of small LDL particles	2 (0.2)
Very large HDL	Concentration of very large HDL particles	4 (0.7)
Large HDL	Concentration of large HDL particles	32 (5.5)[c]
Medium HDL	Concentration of medium HDL particles	1 (0.2)
Small HDL	Concentration of small HDL particles	1 (0.2)
**Lipoprotein particle size**		
VLDL diameter	Mean diameter for VLDL particles	0
LDL diameter	Mean diameter for LDL particles	0
HDL diameter	Mean diameter for HDL particles	0
**Apolipoproteins**		
ApoA1	Apolipoprotein A-1	0
ApoB	Apolipoprotein B	0
ApoB / ApoA1 ratio	Ratio of apolipoprotein B to apolipoprotein A-1	0
**Triglycerides**		
Total triglycerides[a]	Serum total triglycerides	0
VLDL-TG[a]	Triglycerides in VLDL	0
IDL-TG[a]	Triglycerides in IDL	2 (0.3)
LDL-TG	Triglycerides in LDL	0
HDL-TG[a]	Triglycerides in HDL	0
**Cholesterol**		
Total cholesterol	Serum total cholesterol	0
VLDL-C[a]	Total cholesterol in VLDL	0
IDL-C	Total cholesterol in IDL	2 (0.3)
LDL-C	Total cholesterol in LDL	0
HDL-C	Total cholesterol in HDL	0
HDL_2-C	Total cholesterol in HDL_2	2 (0.3)
HDL_3-C	Total cholesterol in HDL_3	0
Esterified cholesterol	Esterified cholesterol	0
Free cholesterol	Free cholesterol	0
**Continues on next page**		

[a]LN-transformed metabolome measure used in all regression analyses because of non-normal distribution.
[b]Lowest value in the data was imputed to individuals if the value was missing (being under the detection limit).
[c]Number of imputed values exceeded the limit of 5%, and all analyses were verified also with non-imputed variables. For these variables coefficients of determination by aerobic fitness would have been slightly larger if imputations were not performed.

© 2019 Kujala UM et al. *JAMA Network Open.*

eTable 2 continues from previous page

Name of variable used in tables	Explanation	Number of imputed values (%)[b]
**Fatty acids**		
Total fatty acids[a]	Total fatty acids	0
Unsaturation degree	Estimated degree of unsaturation	0
Omega-3 FA	Omega-3 fatty acids	0
Omega-3 FA ratio	Ratio of omega-3 fatty acids to total fatty acids	0
Docosahexaenoic acid	22:6, docosahexaenoic acid	0
Polyunsaturated FA	Polyunsaturated fatty acids	0
Omega-6 FA	Omega-6 fatty acids	0
Omega-6 FA ratio	Ratio of omega-6 fatty acids to total fatty acids	0
Linoleic acid	18:2, linoleic acid	0
Saturated FA[a]	Saturated fatty acids	0
Saturated FA ratio	Ratio of saturated fatty acids to total fatty acids	0
Monounsaturated FA[a]	Monounsaturated fatty acids; 16:1, 18:1	0
**Metabolic substrates**		
Glucose	Glucose	0
Glycerol[a]	Glycerol	0
Acetoacetate[a]	Acetoacetate	0
3-hydroxybuturate	3-hydroxybuturate	0
Acetate	Acetate	0
Citrate	Citrate	0
Lactate[a]	Lactate	0
Pyruvate [a]	Pyruvate	0
**Amino acids**		
Isoleucine (BCAA)[a]	Isoleucine	0
Leucine (BCAA)[a]	Leucine	0
Valine (BCAA)	Valine	0
Alanine	Alanine	0
Glutamine	Glutamine	0
Glycine[a]	Glycine	0
Histidine	Histidine	0
Phenylalanine[a]	Phenylalanine	0
Tyrosine	Tyrosine	0
**Miscellaneous**		
Glycoproteins[a]	Glycoprotein acetylation	0
Creatinine	Creatinine	0
Albumin	Albumin	0

[a]LN-transformed metabolome measure used in all regression analyses because of non-normal distribution.
[b]Lowest value in the data was imputed to individuals if the value was missing (being under the detection limit).
[c]Number of imputed values exceeded the limit of 5%, and all analyses were verified also with non-imputed variables. For these variables coefficients of determination by aerobic fitness would have been slightly larger if imputations were not performed.

© 2019 Kujala UM et al. *JAMA Network Open.*

eTable 3. Serum Metabolome Measures by Aerobic Fitness and Muscular Strength Groups in SI Units

Metabolome measure	Aerobic fitness			Muscle strength		
	Low	High	P[a]	Low	High	P[a]
	Median (IQR[b])			Median (IQR[b])		
**Lipoprotein particle concentration**						
Extremely large VLDL, mol/l	1.03e-10 (1.01e-10)	0.538e-10 (0.844e-10)	*P*<2.2e-16	0.683e-10 (0.813e-10)	0.745e-10 (0.871e-10)	*P*=.6371
Very large VLDL, mol/L	4.65e-10 (5.84e-10)	1.74e-10 (3.90e-10)	*P*<2.2e-16	2.63e-10 (4.87e-10)	3.07e-10 (6.14e-10)	*P*=.3985
Large VLDL, mol/L	36.1e-10 (35.8e-10)	19.0e-10 (21.1e-10)	*P*<2.2e-16	25.7e-10 (25.8e-10)	28.0e-10 (33.6e-10)	*P*=.5791
Medium VLDL, mol/L	148e-10 (102e-10)	101e-10 (59e-10)	*P*<2.2e-16	121e-10 (67e-10)	126e-10 (90e-10)	*P*=.5138
Small VLDL, mol/L	264e-10 (107e-10)	204e-10 (75e-10)	*P*<2.2e-16	228e-10 (86e-10)	242e-10 (113e-10)	*P*=.1028
Very small VLDL, mol/L	341e-10 (108e-10)	295e-10 (92e-10)	*P*<2.2e-16	310e-10 (87e-10)	331e-10 (115e-10)	*P*=.0129
IDL, mol/L	918e-10 (325e-10)	815e-10 (265e-10)	*P*=0.0001	843e-10 (282e-10)	913e-10 (345e-10)	*P*=.0104
Large LDL, mol/L	1.50e-7 (0.57e-7)	1.31e-7 (0.47e-7)	*P*<2.2e-16	1.34e-7 (0.50e-7)	1.46e-7 (0.61e-7)	*P*=.0122
Medium LDL, mol/L	1.22e-7 (0.52e-7)	1.04e-7 (0.42e-7)	*P*<2.2e-16	1.08e-7 (0.42e-7)	1.18e-7 (0.52e-7)	*P*=.0181
Small LDL, mol/L	1.43e-7 (0.57e-7)	1.23e-7 (0.47e-7)	*P*<2.2e-16	1.29e-7 (0.47e-7)	1.38e-7 (0.59e-7)	*P*=.0367
Very large HDL, mol/L	3.01e-7 (1.58e-7)	3.87e-7 (2.02e-7)	*P*<2.2e-16	3.50e-7 (1.73e-7)	3.56e-7 (1.82e-7)	*P*=.7024
Large HDL, mol/L	6.33e-7 (4.02e-7)	9.81e-7 (4.37e-7)	*P*<2.2e-16	8.32e-7 (4.20e-7)	8.22e-7 (5.54e-7)	*P*=.6622
Medium HDL, mol/L	15.9e-7 (3.7e-7)	17.2e-7 (2.9e-7)	*P*<2.2e-16	16.9e-7 (3.5e-7)	16.3e-7 (3.4e-7)	*P*=.0538
Small HDL, mol/L	47.3e-7 (5.6e-7)	45.8e-7 (4.3e-7)	*P*=.0093	46.1e-7 (5.4e-7)	45.9e-7 (4.7e-7)	*P*=.5527
**Lipoprotein particle size**						
VLDL diameter, nm	36.8 (1.43)	36.1 (1.47)	*P*<2.2e-16	36.3 (1.52)	36.4 (1.59)	*P*=.9308
LDL diameter, nm	23.5 (0.12)	23.6 (0.12)	*P*=.1361	23.6 (0.12)	23.6 (0.10)	*P*=.4795
HDL diameter, nm	9.71 (0.29)	9.89 (0.29)	*P*<2.2e-16	9.84 (0.25)	9.82 (0.34)	*P*=.4763
**Apolipoproteins**						
ApoA1, g/L	1.27 (0.17)	1.37 (0.17)	*P*<2.2e-16	1.33 (0.17)	1.32 (0.18)	*P*=.5950
ApoB, g/L	0.819 (0.230)	0.676 (0.185)	*P*<2.2e-16	0.707 (0.212)	0.773 (0.252)	*P*=.0019
ApoB / ApoA1 ratio	0.634 (0.193)	0.507 (0.121)	*P*<2.2e-16	0.538 (0.146)	0.582 (0.203)	*P*=.0020
**Continues on the next page**						

In the documentation of concentrations note that 1.03e-10 mol/L = .000000000103 mol/L and in the documentation of *P* values note that *P*<2.2e-16 = *P*<.0000000000000002.2 etc.

[a]*P* for difference between high and low fit group, permutation median test.

[b]IQR is the range between 25% and 75% of observations.

© 2019 Kujala UM et al. *JAMA Network Open.*

eTable 3 continues from the previous page

Metabolome measure	Aerobic fitness			Muscle strength		
	Low	High	*P*[a]	Low	High	*P*[a]
	Median (IQR[b])			Median (IQR[b])		
**Triglycerides**						
Total triglycerides, mmol/L	1.07 (0.529)	0.797 (0.340)	*P*<2.2e-16	0.910 (0.420)	0.943 (0.519)	*P*=.3275
VLDL-TG, mmol/L	0.744 (0.468)	0.500 (0.293)	*P*<2.2e-16	0.612 (0.334)	0.626 (0.440)	*P*=.6346
IDL-TG, mmol/L	0.0878 (0.0299)	0.0755 (0.0197)	*P*<2.2e-16	0.0797 (0.0228)	0.0811 (0.0261)	*P*=.4010
LDL-TG, mmol/L	0.131 (0.052)	0.112 (0.040)	*P*<2.2e-16	0.117 (0.040)	0.120 (0.048)	*P*=.4985
HDL-TG, mmol/L	0.114 (0.037)	0.102 (0.026)	*P*<2.2e-16	0.105 (0.027)	0.108 (0.034)	*P*=.2384
**Cholesterol**						
Total cholesterol, mmol/L	3.69 (0.97)	3.44 (0.92)	*P*=.0010	3.59 (1.01)	3.71 (1.12)	*P*=.1013
VLDL-C, mmol/L	0.604 (0.300)	0.456 (0.178)	*P*<2.2e-16	0.500 (0.222)	0.553 (0.275)	*P*=.0210
IDL-C, mmol/L	0.596 (0.227)	0.521 (0.183)	*P*=.0002	0.541 (0.209)	0.587 (0.241)	*P*=.0129
LDL-C, mmol/L	1.39 (0.67)	1.18 (0.50)	*P*<2.2e-16	1.25 (0.53)	1.35 (0.64)	*P*=.0251
HDL-C, mmol/L	1.09 (0.29)	1.29 (0.26)	*P*<2.2e-16	1.21 (0.26)	1.19 (0.35)	*P*=.3625
HDL_2-C, mmol/L	0.620 (0.284)	0.805 (0.262)	*P*<2.2e-16	0.737 (0.259)	0.706 (0.333)	*P*=.2520
HDL_3-C, mmol/L	0.484 (0.035)	0.486 (0.028)	*P*=.6032	0.485 (0.030)	0.486 (0.027)	*P*=.5689
Esterified cholesterol, mmol/L	2.63 (0.72)	2.47 (0.67)	*P*=.0044	2.57 (0.73)	2.63 (0.80)	*P*=.1905
Free cholesterol, mmol/L	1.06 (0.29)	0.989 (0.25)	*P*=.0019	1.02 (0.27)	1.06 (0.33)	*P*=.1379
**Fatty acids**						
Total fatty acids, mmol/L	9.46 (2.46)	8.30 (2.07)	*P*<2.2e-16	8.60 (2.23)	8,93 (2.80)	*P*=.1065
Unsaturation degree, %	1.21 (0.061)	1.24 (0.050)	*P*<2.2e-16	1.22 (0.057)	1.23 (0.058)	*P*=.0964
Omega-3 FA, mmol/L	0.339 (0.126)	0.303 (0.111)	*P*=.0029	0.296 (0.121)	0.343 (0.154)	*P*<2.2E-16
Omega-3 FA ratio, %	3.60 (0.65)	3.67 (0.72)	*P*=.4171	3.42 (0.75)	3.77 (0.79)	*P*<2.2E-16
Docosahexaenoic acid, mmol/L	0.0879 (0.0396)	0.0796 (0.0336)	*P*=.0132	0.0777 (0.0328)	0.0896 (0.0460)	*P*=.0012
Polyunsaturated FA, mmol/L	3.66 (0.82)	3.38 (0.74)	*P*=.0006	3.46 (0.84)	3.62 (0.89)	*P*=.0366
Omega-6 FA, mmol/L	3.32 (0.74)	3.07 (0.65)	*P*=.0001	3.16 (0.73)	3.27 (0.78)	*P*=.0539
Omega-6 FA ratio, %	35.6 (3.1)	37.3 (2.3)	*P*<2.2e-16	36.5 (2.7)	36.4 (2.9)	*P*=.5098
Linoleic acid, mmol/L	2.87 (0.66)	2.70 (0.60)	*P*=.0010	2.71 (0.67)	2.82 (0.72)	*P*=.0288
Saturated FA, mmol/L	3.21 (0.78)	2.79 (0.67)	*P*<2.2e-16	2.93 (0.72)	3.03 (0.90)	*P*=.0456
Saturated FA ratio, %	34.1 (1.5)	33.7 (1.3)	*P*=.0097	33.9 (1.4)	33.9 (1.5)	*P*=.6681
Monounsaturated FA, mmol/L	2.48 (0.84)	2.14 (0.62)	*P*<2.2e-16	2.24 (0.70)	2.28 (0.81)	*P*=.6093
**Continues on the next page**						

In the documentation of concentrations note that 1.03e-10 mol/L = .000000000103 mol/L and in the documentation of *P* values note that *P*<2.2e-16 = *P*<.0000000000000002.2 etc.

[a]*P* for difference between high and low fit group, permutation median test.

[b]IQR is the range between 25% and 75% of observations.

© 2019 Kujala UM et al. *JAMA Network Open.*

eTable 3 continues from the previous page

Metabolome measure	Aerobic fitness			Muscle strength		
	Low	High	*P*[a]	Low	High	*P*[a]
	Median (IQR[b])			Median (IQR[b])		
**Metabolic substrates**						
Glucose, mmol/L	4.07 (0.44)	3.97 (0.39)	*P*=.0179	3.91 (0.44)	4.02 (0.37)	*P*=.0008
Glycerol, mmol/L	0.0742 (0.0332)	0.0565 (0.0219)	*P*<2.2e-16	0.0666 (0.0202)	0.0616 (0.0267)	*P*=.0052
Acetoacetate, mmol/L	0.0880 (0.1010)	0.0678 (0.0678)	*P*=.0131	0.0950 (0.0950)	0.0658 (0.0605)	*P*<2.2e-16
3-hydroxybuturate, mmol/L	0.165 (0.127)	0.135 (0.105)	*P*<.0008	0.164 (0.132)	0.131 (0.0808)	*P*=.0001
Acetate, mmol/L	0.0417 (0.0148)	0.0416 (0.0119)	*P*=.9598	0.0409 (0.0116)	0.0414 (0.0128)	*P*=.5924
Citrate, mmol/L	0.105 (0.017)	0.105 (0.015)	*P*=.7448	0.105 (0.018)	0.104 (0.015)	*P*=.2535
Lactate, mmol/L	0.904 (0.308)	0.799 (0.333)	*P*=.0006	0.850 (0.299)	0.840 (0.250)	*P*=.6929
Pyruvate, mmol/L	0.0815 (0.0185)	0.0750 (0.0186)	*P*=.0004	0.0778 (0.0192)	0.0745 (0.0199)	*P*=.0294
**Amino acids**						
Isoleucine (BCAA), mmol/L	0.0598 (0.0155)	0.0550 (0.0122)	*P*<2.2e-16	0.0548 (0.0146)	0.0572 (0.0150)	*P*=.1027
Leucine (BCAA), mmol/L	0.0881 (0.0174)	0.0804 (0.0144)	*P*<2.2e-16	0.0813 (0.0189)	0.0831 (0.0166)	*P*=.3342
Valine (BCAA), mmol/L	0.187 (0.034)	0.181 (0.026)	*P*=.0119	0.178 (0.032)	0.185 (0.029)	*P*=.0191
Alanine, mmol/L	0.365 (0.069)	0.347 (0.057)	*P*=.0094	0.342 (0.061)	0.357 (0.054)	*P*=.0043
Glutamine, mmol/L	0.508 (0.113)	0.552 (0.089)	*P*<2.2e-16	0.543 (0.105)	0.517 (0.094)	*P*=.0027
Glycine, mmol/L	0.223 (0.036)	0.233 (0.033)	*P*=.0038	0.227 (0.037)	0.223 (0.030)	*P*=.1361
Histidine, mmol/L	0.0693 (0.0091)	0.0688 (0.0087)	*P*=.4298	0.0691 (0.0082)	0.0685 (0.0093)	*P*=.4862
Phenylalanine, mmol/L	0.0706 (0.0118)	0.0662 (0.0085)	*P*<2.2e-16	0.0651 (0.0099)	0.0687 (0.0085)	*P*<2.2e-16
Tyrosine, mmol/L	0.0546 (0.0119)	0.0493 (0.0101)	*P*<2.2e-16	0.0478 (0.0106)	0.0536 (0.0107)	*P*<2.2e-16
**Miscellaneous**						
Glycoproteins, mmol/L	1.32 (0.24)	1.18 (0.17)	*P*<2.2e-16	1.23 (0.19)	1.23 (0.22)	*P*=.9611
Creatinine, mmol/L	0.0681 (0.0123)	0.0687 (0.0105)	*P*=.5018	0.0663 (0.0108)	0.0701 (0.0117)	*P*=.0002
Albumin, signal area	0.0897 (0.0060)	0.0907 (0.0054)	*P*=.0547	0.0907 (0.0065)	0.0894 (0.0059)	*P*=.0511

In the documentation of concentrations note that 1.03e-10 mol/L = .000000000103 mol/L and in the documentation of *P* values note that *P*<2.2e-16 = *P*<.0000000000000002.2 etc.
[a]*P* for difference between high and low fit group, permutation median test.
[b]IQR is the range between 25% and 75% of observations.

© 2019 Kujala UM et al. *JAMA Network Open.*

eTable 4. Differences in the Medians of Serum Metabolome Measures Between Aerobic Fitness Groups and Between the Muscular Strength Groups Relative to 1 SD of Each Variable[a]

Metabolome measure	Median difference (95% CI) between high fit *vs* low fit group relative to SD, *P* value[b]	
	**High *vs* low aerobic fitness**	**High *vs* low muscle strength**
**Lipoprotein particle concentration**		
Extremely large VLDL	-0.344 (-0.437 to -0.267), *P*<.001	-0.051 (-0.087 to .0179), *P*=.64
Very large VLDL	-0.375 (-0.475 to -0.290), *P*<.001	0.054 (-0.077 to 0.197), *P*=.40
Large VLDL	-0.417 (-0.529 to -0.313), *P*<.001	0.048 (-0.104 to 0.209), *P*=.58
Medium VLDL	-0.497 (-0.662 to -0.357), *P*<.001	0.070 (-0.115 to 0.215), *P*=.51
Small VLDL	-0.674 (-0.826 to -0.487), *P*<.001	0.151 (-0.052 to 0.366), *P*=.10
Very small VLDL	-0.604 (-0.849 to -0.382), *P*<.001	0.272 (0.046 to 0.476), *P*=.01
IDL	-0.468 (-0.637 to -0.209), *P*=.001	0.272 (0.087 to 0.484), *P*=.01
Large LDL	-0.464 (-0.658 to -0.266), *P*<.001	0.257 (0.082 to 0.529), *P*=.01
Medium LDL	-0.487 (-0.727 to -0.289), *P*<.001	0.259 (0.019 to 0.488), *P*=.02
Small LDL	-0.461 (-0.755 to -0.230), *P*<.001	0.246 (-0.024 to 0.433), *P*=.04
Very large HDL	0.616 (0.418 to 0.940), *P*<.001	0.008 (-0.245 to 0.275), *P*=.70
Large HDL	0.890 (0.691 to 1.153), *P*<.001	-0.104 (-0.276 to 0.187), *P*=.66
Medium HDL	0.530 (0.324 to 0.673), *P*<.001	-0.248 (-0.448 to -0.004), *P*=.05
Small HDL	-0.383 (-0.651 to -0.092), *P*=.009	-0.083 (-0.304 to 0.156), *P*=.55
**Lipoprotein particle size**		
VLDL diameter	-0.538 (-0.770 to -0.348), *P*<.001	0.039 (-0.246 to 0.230), *P*=.93
LDL diameter	0.121 (-0.059 to 0.177), *P*=.14	-0.071 (-0.235 to 0.050), *P*=.48
HDL diameter	0.839 (0.569 to 1.087), *P*<.001	-0.092 (-0.373 to 0.133), *P*=.48
**Apolipoproteins**		
ApoA1	0.695 (0.450 to 0.872), *P*<.001	-0.046 (-0.382 to 0.154), *P*=.60
ApoB	-0.798 (-1.054 to -0.598), *P*<.001	0.346 (0.130 to 0.584), *P*=.002
ApoB / ApoA1 ratio	-0.880 (-1.075 to -0.671), *P*<.001	0.322 (0.102 to 0.516), *P*=.002
**Triglycerides**		
Total triglycerides	-0.523 (-0.645 to -0.344), *P*<.001	0.062 (-0.072 to 0.199), *P*=.33
VLDL-TG	-0.483 (-0.615 to -0.353), *P*<.001	0.055 (-0.153 to 0.176), *P*=.63
IDL-TG	-0.589 (-0.827 to -0.364), *P*<.001	0.073 (-0.132 to 0.255), *P*=.40
LDL-TG	-0.519 (-0.765 to -0.262), *P*<.001	0.072 (-0.163 to 0.354), *P*=.50
HDL-TG	-0.431 (-0.661 to -0.239), *P*<.001	0.147 (-0.122 to 0.323), *P*=.24
**Cholesterol**		
Total cholesterol	-0.292 (-0.607 to -0.142), *P*=.001	0.149 (-0.038 to 0.370), *P*=.10
VLDL-C	-0.695 (-0.868 to -0.487), *P*<.001	0.266 (0.026 to 0.427), *P*=.02
IDL-C	-0.458 (-0.664 to -0.267), *P*=.002	0.262 (0.033 to 0.475), *P*=.01
LDL-C	-0.444 (-0.643 to -0.217), *P*<.001	0.263 (-0.037 to 0.444), *P*=.03
HDL-C	0.814 (0.559 to 1.018), *P*<.001	-0.139 (-0.344 to 0.211), *P*=.36
HDL_2-C	0.829 (0.595 to 1.047), *P*<.001	-0.157 (-0.394 to 0.140), *P*=.25
HDL_3-C	0.080 (-0.139 to 0.278), *P*=.60	0.062 (-0.157 to 0.219), *P*=.57
Esterified cholesterol	-0.287 (-0.496 to -0.049), *P*=.004	0.139 (-0.096 to 0.313), *P*=.19
Free cholesterol	-0.335 (-0.567 to -0.108), *P*=.002	0.188 (-0.112 to 0.381), *P*=.14
**Continues on the next page**		

[a]This data has been used to construct the manuscript figures 2 and 3. Differences in medians and their 95% CIs calculated by bootstrapping. Values are per 1SD of the variable's distribution among all the 580 participants and positive values indicate higher level in high fit group.

[b]*P* values are for difference between high and low fit group, by permutation median test, *P* significant at *P*≤.002 (see statistical methods). For more exact *P* values see eTable 3 in this Supplement.

© 2019 Kujala UM et al. *JAMA Network Open.*

eTable 4 continues from the previous page

Metabolome measure	Median difference (95% CI) between high fit *vs* low fit group relative to SD, *P* value[b]	
	**High *vs* low aerobic fitness**	**High *vs* low muscle strength**
**Fatty acids**		
Total fatty acids	-0.605 (-0.877 to -0.423), *P*<.001	0.183 (-0.033 to 0.347), *P*=.11
Unsaturation degree	0.588 (0.417 to 0.814), *P*<.001	0.167 (-0.062 to 0.393), *P*=.10
Omega-3 FA	-0.323 (-0.632 to -0.080), *P*=.003	0.460 (0.240 to 0.717), *P*<.001
Omega-3 FA ratio	0.143 (-0.158 to 0.402), *P*=.42	0.597 (0.284 to 0.798), *P*<.001
Docosahexaenoic acid	-0.264 (-0.481 to -0.033), *P*=.01	0.381 (0.124 to 0.644), *P*=.001
Polyunsaturated FA	-0.447 (-.0643 to -0.171), *P*<.001	0.279 (-0.008 to 0.480), *P*=.04
Omega-6 FA	-0.442 (-0.700 to -0.202), *P*<.001	0.228 (-0.019 to 0.399), *P*=.05
Omega-6 FA ratio	0.683 (0.463 to 0.844), *P*<.001	-0.074 (-0.259 to 0.219), *P*=.51
Linoleic acid	-0.362 (-0.676 to -0.079), *P*=.001	0.230 (-0.025 to 0.455), *P*=.03
Saturated FA	-0.647 (-0.890 to -0.449), *P*<.001	0.183 (-0.037 to 0.346), *P*=.05
Saturated FA ratio	-0.285 (-0.537 to -0.103), *P*=.01	0.055 (-0.192 to 0.256), *P*=.67
Monounsaturated FA	-0.570 (-0.787 to -0.347), *P*<.001	0.060 (-0.200 to 0.261), *P*=.61
**Metabolic substrates**		
Glucose	-0.270 (-0.578 to 0.045), *P*=.018	0.333 (0.163 to 0.534), *P*<0.001
Glycerol	-0.636 (-0.806 to -0.480), *P*<.001	-0.236 (-0.398 to -0.056), *P*=.01
Acetoacetate	-0.224 (-0.365 to -0.060), *P*=.01	-0.332 (-0.514 to -0.207), *P*<.001
3-hydroxybuturate	-0.204 (-0.314 to -0.094), *P*<.001	-0.229 (-0.355 to -0.088), *P*<.001
Acetate	-0.008 (-0.270 to 0.206), *P*=.96	0.046 (-0.138 to 0.240), *P*=.59
Citrate	0.030 (-0.190 to 0.268), *P*=.74	-0.127 (-0.357 to 0.108), *P*=.25
Lactate	-0.344 (-0.559 to -0.187), *P*<.001	-0.047 (-0.248 to 0.166), *P*=.69
Pyruvate	-0.362 (-0.586 to -0.141), *P*<.001	-0.193 (-0.418 to -0.019), *P*=.03
**Amino acids**		
Isoleucine (BCAA)	-0.374 (-0.554 to -0.164), *P*<.001	0.138 (-0.057 to 0.367), *P*=.10
Leucine (BCAA)	-0.546 (-0.716 to -0.336), *P*<.001	0.118 (-0.147 to 0.381), *P*=.33
Valine (BCAA)	-0.301 (-0.498 to -0.039), *P*=.01	0.256 (0.055 to 0.529), *P*=.02
Alanine	-0.328 (-0.566 to -0.048), *P*=.01	0.302 (0.117 to 0.522), *P*=.004
Glutamine	0.574 (0.358 to 0.791), *P*<.001	-0.325 (-0.634 to -0.092), *P*=.003
Glycine	0.361 (0.108 to 0.574), *P*=.004	-0.183 (-0.383 to 0.074), *P*=.14
Histidine	-0.080 (-0.330 to 0.134), *P*=.43	-0.093 (-0.367 to 0.176), *P*=.49
Phenylalanine	-0.539 (-0.708 to -0.324), *P*<.001	0.470 (0.240 to 0.703), *P*<.001
Tyrosine	-0.554 (-0.783 to -0.350), *P*<.001	0.693 (0.489 to 0.914), *P*<.001
**Miscellaneous**		
Glycoproteins	-0.776 (-0.946 to -0.616), *P*<.001	0.009 (-0.172 to 0.229), *P*=.96
Creatinine	0.108 (-0.173 to 0.304), *P*=.50	0.420 (0.190 to 0.744), *P*=.002
Albumin	0.234 (0.007 to 0.440), *P*=.06	-0.278 (-0.496 to 0.025), *P*=.05

[a]This data has been used to construct the manuscript figures 2 and 3. Differences in medians and their 95% CIs calculated by bootstrapping. Values are per 1SD of the variable's distribution among all the 580 participants and positive values indicate higher level in high fit group.
[b]*P* values are for difference between high and low fit group, by permutation median test, *P* significant at *P*≤.002 (see statistical methods). For more exact *P* values see eTable 3 in this Supplement.

© 2019 Kujala UM et al. *JAMA Network Open.*

eTable 5. Linear Regression Analysis of the Age-Adjusted Association of Aerobic Fitness With Serum Metabolome Measures

Metabolome measure	β[a]	*P*[a]	R^{2b}(%) age and aerobic fitness	R^{2c} (%) age	ΔR^{2d} (%)
**Lipoprotein particle concentration**					
Extremely large VLDL	-0.05188	1.797e-09	8.24	2.30	5.94
Very large VLDL	-0.08973	1.106e-09	7.84	1.70	6.14
Large VLDL	-0.04817	9.074e-13	9.88	1.54	8.34
Medium VLDL	-0.02040	3.567e-17	15.38	4.29	11.09
Small VLDL	-0.01324	2.982e-18	20.92	9.79	11.16
Very small VLDL	-2.334e-10	1.344e-11	23.96	17.68	6.28
IDL	-4.970e-10	2.272e-06	16.42	13.12	3.30
Large LDL	-8.819e-10	2.599e-06	15.93	12.64	3.29
Medium LDL	-8.058e-10	7.696e-07	16.85	13.25	3.60
Small LDL	-8.756e-10	1.341e-06	16.91	13.47	3.44
Very large HDL	5.535e-09	5.856e-17	11.44	0.00	11.44
Large HDL	1.740e-08	8.241e-27	21.78	4.52	17.26
Medium HDL	5.757e-09	3.093e-06	9.53	6.06	3.47
Small HDL	-7.572e-09	2.978e-05	3.49	0.53	2.96
**Lipoprotein particle size**					
VLDL diameter	-0.04265	9.185e-13	8.72	0.27	8.45
LDL diameter	0.001505	0.03579	1.95	1.20	0.75
HDL diameter	0.01089	2.305e-27	21.01	3.16	17.85
**Apolipoproteins**					
ApoA1	0.003910	9.538e-10	6.38	0.11	6.27
ApoB	-0.006043	9.160e-15	22.41	13.89	8.52
ApoB / ApoA1 ratio	-0.006371	4.773e-26	29.02	13.89	15.13
**Triglycerides**					
Total triglycerides	-0.01587	3.584e-19	18.40	6.24	12.16
VLDL-TG	-0.02053	9.226e-19	16.12	3.93	12.19
IDL-TG	-0.007359	1.327e-12	21.41	14.24	7.17
LDL-TG	-0.9415e-3	7.291e-10	23.81	18.63	5.18
HDL-TG	-0.004996	1.631e-06	10.50	6.86	3.64
**Cholesterol**					
Total cholesterol	-0.009733	6.238e-03	11.84	10.68	1.16
VLDL-C	-0.01282	1.040e-14	20.61	11.93	8.68
IDL-C	-0.003208	1.698e-05	14.24	11.44	2.80
LDL-C	-0.008730	1.776e-05	14.60	11.82	2.78
HDL-C	0.009940	3.613e-19	14.89	2.20	12.69
HDL_2-C	0.009601	8.635e-21	17.20	3.63	13.57
HDL_3-C	0.3036e-03	1.853e-02	7.49	6.59	0.90
Esterified cholesterol	-0.006806	8.161e-03	11.29	10.20	1.09
Free cholesterol	-0.002926	3.255e-03	13.10	11.79	1.31
**Continues on the next page**					

[a]Unstandardised regression coefficient (β) and *P* value for aerobic fitness in the model adjusted for age. In the documentation of regression coefficient note that -0.2334e-10 means -0.00000000002334 and in the documentation of *P* values note that *P*=1.797e-09 means *P*=.000000001797 etc.
[b]R^2 is the coefficient of determination for the model with age and aerobic fitness (independent variables) in relation to each metabolome measure (dependent variable).
[c]R^2 is the total coefficient of determination for the model with age as independent variable.
[d]ΔR^2 is the difference of the coefficients of determination between the models [(age and aerobic fitness) – (age)] = additional explanation rate of aerobic fitness after age in the model.

© 2019 Kujala UM et al. *JAMA Network Open.*

eTable 5 continues from the previous page

Metabolome measure	β[a]	*P*[a]	R^{2b}(%) age and aerobic fitness	R^{2c} (%) age	ΔR^{2d} (%)
**Fatty acids**					
Total fatty acids	-0.005076	1.204e-08	17.24	12.43	4.81
Unsaturation degree	0.001872	2.466e-17	13.42	1.91	11.51
Omega-3 FA	-0.9001e-03	4.363e-02	14.42	13.81	0.61
Omega-3 FA ratio	0.01064	1.153e-04	7.40	4.97	2.43
Docosahexaenoic acid	-0.2588e-03	6.825e-02	13.62	13.12	0.40
Polyunsaturated FA	-0.008814	2.102e-03	13.62	12.19	1.43
Omega-6 FA	-0.007915	1.398e-03	12.98	11.42	1.56
Omega-6 FA ratio	0.09830	6.357e-17	14.59	3.54	11.05
Linoleic acid	-0.006417	3.246e-03	11.48	10.13	1.35
Saturated FA	-0.005494	2.109e-09	17.48	12.16	5.32
Saturated FA ratio	-0.01502	0.01479	1.12	0.10	1.02
Monounsaturated FA	-0.008606	1.570e-13	17.96	9.79	8.17
**Metabolic substrates**					
Glucose	-0.002866	6.482e-02	5.61	5.05	0.56
Glycerol	-0.01529	4.695e-27	18.54	0.35	18.19
Acetoacetate	-0.01315	1.849e-05	3.30	0.17	3.13
3-hydroxybuturate	-0.01192	2.228e-06	3.92	0.12	3.80
Acetate	-2.026e-05	0.9847	0.29	0.29	0.00
Citrate	-4.275e-05	0.4564	0.16	0.06	0.10
Lactate	-0.003868	0.002299	2.38	0.79	1.59
Pyruvate	-0.004560	1.729e-06	3.94	0.00	3.94
**Amino acids**					
Isoleucine (BCAA)	-0.006418	1.823e-10	6.81	0.00	6.81
Leucine (BCAA)	-0.004880	1.059e-10	7.27	0.31	6.96
Valine (BCAA)	-0.3956e-03	6.345e-04	4.01	2.05	1.96
Alanine	-0.7044e-03	1.616e-03	3.24	1.56	1.68
Glutamine	0.001810	3.190e-08	21.67	17.36	4.31
Glycine	0.001622	4.708e-03	1.44	0.06	1.38
Histidine	-2.981e-05	0.3378	0.89	0.74	0.15
Phenylalanine	-3.556e-03	4.253e-11	7.29	0.02	7.27
Tyrosine	-1.780e-04	1.842e-05	3.25	0.12	3.13
**Miscellaneous**					
Glycoproteins	-0.006786	2.569e-29	19.81	0.16	19.65
Creatinine	7.114e-05	8.028e-02	1.55	1.02	0.53
Albumin	1.923e-05	0.3318	20.16	20.03	0.13

[a]Unstandardised regression coefficient (β) and *P* value for aerobic fitness in the model adjusted for age. In the documentation of regression coefficient note that -0.2334e-10 means -0.00000000002334 and in the documentation of *P* values note that *P*=1.797e-09 means *P*=.000000001797 etc.
[b]R^2 is the coefficient of determination for the model with age and aerobic fitness (independent variables) in relation to each metabolome measure (dependent variable).
[c]R^2 is the total coefficient of determination for the model with age as independent variable.
[d]ΔR^2 is the difference of the coefficients of determination between the models [(age and aerobic fitness) – (age)] = additional explanation rate of aerobic fitness after age in the model.

© 2019 Kujala UM et al. *JAMA Network Open.*

eTable 6. Linear Regression Analysis of the Age-Adjusted Association of Maximal Muscular Strength With Serum Metabolome Measures

Metabolome measure	β[a]	*P*[a]	R^{2b} (%) age and maximal strength	R^{2c} (%) age	ΔR^{2d} (%) (age+abs strength – age)
**Lipoprotein particle concentration**					
Extremely large VLDL	2.217e-04	0.7625	2.31	2.30	0.01
Very large VLDL	-5.224e-04	0.6768	1.74	1.70	0.04
Large VLDL	-0.0003460	0.5489	1.60	1.54	0.06
Medium VLDL	1.114e-04	0.5940	4.34	4.29	0.05
Small VLDL	1.370e-04	0.2972	9.96	9.79	0.17
Very small VLDL	5.868e-12	4.617e-02	18.24	17.68	0.56
IDL	1.308e-11	0.1409	13.45	13.12	0.33
Large LDL	2.451e-11	0.1220	13.00	12.64	0.36
Medium LDL	2.093e-11	0.1290	13.59	13.25	0.34
Small LDL	2.172e-11	0.1561	13.77	13.47	0.30
Very large HDL	1.252e-11	0.8264	0.00	0.00	0.00
Large HDL	-1.518e-10	0.2877	4.71	4.52	0.19
Medium HDL	-3.005e-10	3.846e-03	7.41	6.06	1.35
Small HDL	-3.746e-10	1.414e-02	1.57	0.53	1.04
**Lipoprotein particle size**					
VLDL diameter	-1.197e-04	0.8149	0.28	0.27	0.01
LDL diameter	-1.002e-04	9.560e-02	1.67	1.20	0.47
HDL diameter	-3.588e-05	0.6853	3.19	3.16	0.03
**Apolipoproteins**					
ApoA1	-5.588e-05	0.3042	0.29	0.11	0.18
ApoB	1.329e-04	4.696e-02	14.48	13.89	0.59
ApoB / ApoA1 ratio	1.235e-04	1.955e-02	14.70	13.89	0.81
**Triglycerides**					
Total triglycerides	9.524e-05	0.5356	6.31	6.24	0.07
VLDL-TG	7.050e-05	0.7259	3.96	3.93	0.03
IDL-TG	6.756e-05	0.4468	14.33	14.24	0.09
LDL-TG	1.423e-05	0.2740	18.80	18.63	0.17
HDL-TG	8.772e-05	0.3.195	7.02	6.86	0.16
**Cholesterol**					
Total cholesterol	3.700e-04	0.2156	10.92	10.68	0.24
VLDL-C	2.284e-04	0.1084	12.32	11.93	0.39
IDL-C	9.886e-05	0.1160	11.82	11.44	0.38
LDL-C	2.577e-04	0.1331	12.16	11.82	0.34
HDL-C	-1.570e-04	0.1027	2.66	2.20	0.46
HDL_2-C	-1.418e-04	0.1119	4.05	3.63	0.42
HDL_3-C	-1.055e-05	0.3293	6.75	6.59	0.16
Esterified cholesterol	2.433e-04	0.2602	10.40	10.20	0.20
Free cholesterol	1.272e-04	0.1278	12.14	11.79	0.35
**Continues on the next page**					

[a]Unstandardised regression coefficient (β) and *P* value for muscular strength in the model adjusted for age. In the documentation of regression coefficient note that 2.217e-04 means -0.0002217 and in the documentation of *P* values note that *P*=4.617e-02 means *P*=0.04617 etc.
[b]R^2 is the total coefficient of determination for the model with age and maximal strength (independent variables) in relation to each metabolome measure (dependent variable).
[c]R^2 is the total coefficient of determination for the model with age as independent variable.
[d]ΔR^2 is the difference of the coefficients of determination between the models [(age and muscular strength) – (age)] = additional explanation rate of muscular strength after age in the model.

© 2019 Kujala UM et al. *JAMA Network Open.*

eTable 6 continues from the previous page

Metabolome measure	β[a]	*P*[a]	R^{2b} (%) age and maximal strength	R^{2c} (%) age	ΔR^{2d} (%) (age+abs strength – age)
**Fatty acids**					
Total fatty acids	6.748e-05	0.3741	12.55	12.43	0.12
Unsaturation degree	4.129e-05	3.084e-02	2.70	1.91	0.79
Omega-3 FA	1.312e-04	4.461e-04	15.64	13.81	1.83
Omega-3 FA ratio	0.001100	1.981e-06	8.64	4.97	3.67
Docosahexaenoic acid	3.720e-05	1.774e-03	14.59	13.12	1.47
Polyunsaturated FA	3.662e-04	0.1298	12.54	12.19	0.35
Omega-6 FA	2.349e-04	0.2614	11.62	11.42	0.20
Omega-6 FA ratio	-4.990e-04	0.6243	3.58	3.54	0.04
Linoleic acid	1.207e-04	0.5118	10.20	10.13	0.07
Saturated FA	8.618e-05	0.2710	12.35	12.16	0.19
Saturated FA ratio	7.165e-04	0.1674	0.42	0.10	0.32
Monounsaturated FA	1.728e-05	0.8633	9.80	9.79	0.01
**Metabolic substrates**					
Glucose	2.877e-04	2.684e-02	5.85	5.05	0.80
Glycerol	-3.591e-04	3.944e-03	1.77	0.35	1.42
Acetoacetate	-0.001149	8.444e-06	3.55	0.17	3.38
3-hydroxybuturate	-9.286e-04	1.102e-05	3.41	0.12	3.29
Acetate	-1.211e-06	0.9880	0.29	0.29	0.00
Citrate	-9.332e-06	5.197e-02	0.72	0.06	0.66
Lactate	-7.816e-05	0.4638	0.89	0.79	0.10
Pyruvate	-0.0001797	2.552e-02	0.87	0.00	0.87
**Amino acids**					
Isoleucine (BCAA)	1.374e-04	0.1089	0.45	0.00	0.45
Leucine (BCAA)	1.263e-04	4.959e-02	0.97	0.31	0.66
Valine (BCAA)	2.567e-05	8.248e-03	3.23	2.05	1.18
Alanine	3.840e-05	4.064e-02	2.27	1.56	0.71
Glutamine	-5.684e-05	4.041e-02	17.97	17.36	0.61
Glycine	-1.037e-04	3.115e-02	0.86	0.06	0.80
Histidine	1.289e-06	0.6209	0.78	0.74	0.04
Phenylalanine	1.479e-04	1.246e-03	1.82	0.02	1.80
Tyrosine	1.853e-05	9.059e-08	4.95	0.12	4.83
**Miscellaneous**					
Glycoproteins	-5.390e-06	0.9196	0.17	0.16	0.01
Creatinine	1.972e-05	4.418e-09	6.76	1.02	5.74
Albumin	-3.127e-06	5.929e-02	20.52	20.03	0.49

[a]Unstandardised regression coefficient (β) and *P* value for muscular strength in the model adjusted for age. In the documentation of regression coefficient note that 2.217e-04 means -0.0002217 and in the documentation of *P* values note that *P*=4.617e-02 means *P*=0.04617 etc.
[b]R^2 is the total coefficient of determination for the model with age and maximal strength (independent variables) in relation to each metabolome measure (dependent variable).
[c]R^2 is the total coefficient of determination for the model with age as independent variable.
[d]ΔR^2 is the difference of the coefficients of determination between the models [(age and muscular strength) – (age)] = additional explanation rate of muscular strength after age in the model.

© 2019 Kujala UM et al. *JAMA Network Open.*

eTable 7. Linear Regression Analysis of The Age-, Education-, Smoking-, Use of Alcohol-, and Diet-Adjusted Association of Aerobic Fitness With Serum Metabolome Measures

Metabolome measure	β[a]	*P*[a]	R^{2b} (%)	R^{2c} (%)	ΔR^{2d} (%)
**Lipoprotein particle concentration**					
Extremely large VLDL	-0.04819	9.217e-08	9.27	4.54	4.73
Very large VLDL	-0.08268	7.594e-08	9.19	4.39	4.80
Large VLDL	-0.04346	4.801e-10	12.13	5.86	6.28
Medium VLDL	-0.01887	5.554e-14	17.14	8.36	8.78
Small VLDL	-0.01239	4.394e-15	22.27	13.27	9.00
Very small VLDL	-2.385e-10	3.684e-11	24.37	18.24	6.13
IDL	-5.410e-10	7.849e-07	17.24	13.56	3.67
Large LDL	-9.509e-10	1.206e-06	16.56	12.99	3.58
Medium LDL	-8.522e-10	5.735e-07	17.36	13.60	3.76
Small LDL	-9.217e-10	1.139e-06	17.51	13.96	3.55
Very large HDL	4.926e-09	4.283e-13	13.97	5.53	8.43
Large HDL	1.688e-08	1.531e-23	23.18	8.21	14.97
Medium HDL	6.797e-09	1.006e-07	14.07	9.62	4.45
Small HDL	-5.100e-09	5.626e-03	10.18	8.94	1.23
**Lipoprotein particle size**					
VLDL diameter	-0.03820	6.470e-10	10.94	4.68	6.27
LDL diameter	0.001292	8.062e-02	6.83	6.32	0.51
HDL diameter	0.01019	7.062e-23	23.35	8.90	14.45
**Apolipoproteins**					
ApoA1	0.003920	3.387e-09	9.57	3.76	5.81
ApoB	-0.006178	3.978e-14	22.91	14.65	8.26
ApoB / ApoA1	-0.006492	1.095e-24	29.69	15.20	14.49
**Triglycerides**					
Total triglycerides	-0.01479	9.575e-16	20.07	10.34	9.73
VLDL-TG	-0.01894	3.219e-15	18.17	8.60	9.57
IDL-TG	-0.007108	5.800e-11	21.60	15.38	6.22
LDL-TG	-0.0009358	4.545e-09	23.84	19.04	4.81
HDL-TG	-0.004702	1.630e-05	11.09	8.10	2.99
**Cholesterol**					
Total cholesterol	-0.01089	3.322e-03	12.95	11.60	1.35
VLDL-C	-0.01256	4.532e-13	21.30	13.60	7.70
IDL-C	-0.003574	4.255e-06	15.19	11.94	3.25
LDL-C	-0.009480	8.203e-06	15.28	12.23	3.05
HDL-C	0.009860	1.402e-17	17.19	5.72	11.47
HDL_2-C	0.0095218	4.874e-19	19.34	7.07	12.27
HDL_3-C	0.0003032	2.408e-02	9.52	8.70	0.82
Esterified cholesterol	-0.007623	4.469e-03	12.44	11.17	1.27
Free cholesterol	-0.003263	1.623e-03	14.12	12.58	1.53
**Continues on the next page**					

[a]Unstandardised regression coefficient (β) and *P* value for aerobic fitness in the model adjusted for age, education, smoking, use of alcohol, and diet (fruits and vegetables, meat, fish, and chicken). In the documentation of regression coefficient note that -2.385e-10 means -0.0000000002385 and in the documentation of *P* values note that *P*=9.217e-08 means *P*=0.00000009217 etc.
[b]R^2 is the coefficient of determination for the model with age, aerobic fitness, education, smoking, use of alcohol, and diet (fruits and vegetables, meat, fish, and chicken) (independent variables) in relation to each metabolome measure (dependent variable).
[c]R^2 is the coefficient of determination for the model with age, education, smoking, use of alcohol, and diet (fruits and vegetables, meat, fish, and chicken) as independent variables.
[d]ΔR^2 is the difference of the coefficients of determination between the models [(age, aerobic fitness, education, smoking, use of alcohol, and diet (fruits and vegetables, meat, fish, and chicken)) – (age, education, smoking, use of alcohol, and diet (fruits and vegetables, meat, fish, and chicken))] = additional explanation rate of aerobic fitness after age, education, smoking, use of alcohol, and diet (fruits and vegetables, meat, fish, and chicken) in the model.

© 2019 Kujala UM et al. *JAMA Network Open.*

eTable 7 continues from the previous page

Metabolome measure	β[a]	*P*[a]	R^{2b} (%)	R^{2c} (%)	ΔR^{2d} (%)
**Fatty acids**					
Total fatty acids	-0.005047	5.998e-08	18.47	14.07	4.39
Unsaturation degree	0.001644	3.349e-13	17.27	9.05	8.22
Omega-3 FA	-0.001255	6.526e-03	16.43	15.32	1.11
Omega-3 FA ratio	0.006272	2.388e-02	13.95	13.16	0.79
Docosahexaenoic acid	-3.813e-04	8.894e-03	16.17	15.13	1.03
Polyunsaturated FA	-0.009845	9.953e-04	14.70	13.04	1.67
Omega-6 FA	-0.008590	8.990e-04	14.04	12.33	1.71
Omega-6 FA ratio	0.08983	1.738e-13	16.64	8.15	8.49
Linoleic acid	-0.006889	2.508e-03	12.50	11.06	1.44
Saturated FA	-0.005551	7.126e-09	18.79	13.78	5.01
Saturated FA ratio	-0.01827	0.004601	3.33	1.93	1.40
Monounsaturated FA	-0.007949	5.209e-11	19.97	13.56	6.41
**Metabolic substrates**					
Glucose	-0.002465	0.1283	8.10	7.72	0.38
Glycerol	-0.01499	2.7531e-24	20.19	4.03	16.17
Acetoacetate	-0.01241	9.033e-05	6.99	4.41	2.58
3-hydroxybuturate	-0.01108	1.748e-05	9.48	6.45	3.02
Acetate	-0.0004851	0.6545	1.98	1.95	0.03
Citrate	-6.311e-05	0.2867	3.02	2.83	0.20
Lactate	-0.004169	0.001540	4.28	2.55	1.73
Pyruvate	-0.003984	5.643e-05	5.97	3.18	2.79
**Amino acids**					
Isoleucine (BCAA)	-6.689e-03	1.647e-10	7.85	0.90	6.95
Leucine (BCAA)	-0.005022	1.628e-10	8.91	2.04	6.88
Valine	-4.818e-04	6.588e-05	5.78	3.07	2.71
Alanine	-0.0007703	9.161e-04	5.04	3.16	1.88
Glutamine	0.002007	4.546e-09	22.72	17.80	4.92
Glycine	0.001927	1.363e-03	2.90	1.11	1.79
Histidine	-1.714e-05	0.5960	2.80	2.76	0.05
Phenylalanine	-0.003496	3.955e-10	10.52	4.06	6.46
Tyrosine	-2.062e-04	1.918e-06	5.85	1.97	3.88
**Miscellaneous**					
Glucoproteins	-0.006350	1.763e-24	22.11	6.21	15.90
Creatinine	1.736e-05	0.6772	5.36	5.33	0.03
Albumine	2.242e-05	0.2739	23.01	22.85	0.16

[a]Unstandardised regression coefficient (β) and *P* value for aerobic fitness in the model adjusted for age, education, smoking, use of alcohol, and diet (fruits and vegetables, meat, fish, and chicken). In the documentation of regression coefficient note that -2.385e-10 means -0.0000000002385 and in the documentation of *P* values note that *P*=9.217e-08 means *P*=0.00000009217 etc.
[b]R^2 is the coefficient of determination for the model with age, aerobic fitness, education, smoking, use of alcohol, and diet (fruits and vegetables, meat, fish, and chicken) (independent variables) in relation to each metabolome measure (dependent variable).
[c]R^2 is the coefficient of determination for the model with age, education, smoking, use of alcohol, and diet (fruits and vegetables, meat, fish, and chicken) as independent variables.
[d]ΔR^2 is the difference of the coefficients of determination between the models [(age, aerobic fitness, education, smoking, use of alcohol, and diet (fruits and vegetables, meat, fish, and chicken)) – (age, education, smoking, use of alcohol, and diet (fruits and vegetables, meat, fish, and chicken))] = additional explanation rate of aerobic fitness after age, education, smoking, use of alcohol, and diet (fruits and vegetables, meat, fish, and chicken) in the model.

© 2019 Kujala UM et al. *JAMA Network Open.*

eTable 8. Linear Regression Analysis of the Age-, Education-, Smoking-, Use of Alcohol-, and Diet-Adjusted Association of Maximal Muscular Strength With Serum Metabolome Measures

Metabolome measure	β[a]	*P*[a]	R^{2b} (%)	R^{2c} (%)	ΔR^{2d} (%)
**Lipoprotein particle concentration**					
Extremely large VLDL	7.638e-04	0.3223	4.70	4.54	0.17
Very large VLDL	6.314e-04	0.6314	4.43	4.39	0.04
Large VLDL	3.542e-04	0.5550	5.91	5.86	0.06
Medium VLDL	3.694e-04	8.894e-02	8.83	8.36	0.47
Small VLDL	3.005e-04	2.813e-02	14.01	13.27	0.74
Very small VLDL	7.674e-12	1.321e-02	19.13	18.24	0.89
IDL	1.470e-11	0.1157	13.94	13.56	0.38
Large LDL	2.774e-11	9.687e-02	13.41	12.99	0.43
Medium LDL	2.487e-11	8.727e-02	14.04	13.60	0.45
Small LDL	2.633e-11	0.1032	14.37	13.96	0.41
Very large HDL	-6.897e-11	0.2404	5.76	5.53	0.23
Large HDL	-3.128e-10	3.549e-02	8.93	8.21	0.72
Medium HDL	-2.688e-10	1.360e-02	10.60	9.62	0.97
Small HDL	-1.412e-10	0.3659	9.07	8.94	0.13
**Lipoprotein particle size**					
VLDL diameter	4.651e-04	0.3813	4.81	4.68	0.13
LDL diameter	-1.185e-04	5.781e-02	6.92	6.32	0.60
HDL diameter	-1.600e-04	7.912e-02	9.40	8.90	0.50
**Apolipoproteins**					
ApoA1	-8.196e-05	0.1495	4.12	3.76	0.36
ApoB	1.728e-04	1.444e-02	15.55	14.65	0.90
ApoB / ApoA1	1.625e-04	3.586e-03	16.47	15.20	1.27
**Triglycerides**					
Total triglycerides	2.881e-04	7.146e-02	10.86	10.34	0.52
VLDL-TG	3.351e-04	0.1078	9.02	8.60	0.42
IDL-TG	1.407e-04	0.1318	15.72	15.38	0.34
LDL-TG	2.220e-05	0.1046	19.42	19.04	0.38
HDL-TG	1.530e-04	9.939e-02	8.54	8.10	0.44
**Cholesterol**					
Total cholesterol	3.951e-04	0.2087	11.85	11.60	0.25
VLDL-C	3.432e-04	2.191e-02	14.40	13.60	0.80
IDL-C	1.042e-04	0.1156	12.32	11.94	0.39
LDL-C	2.872e-04	0.1126	12.62	12.23	0.39
HDL-C	-2.324e-04	2.083e-02	6.61	5.72	0.89
HDL_2-C	-2.165e-04	2.025e-02	7.96	7.07	0.89
HDL_3-C	-1.133e-05	0.3195	8.86	8.70	0.16
Esterified cholesterol	2.597e-04	0.2532	11.37	11.17	0.21
Free cholesterol	1.357e-04	0.1221	12.95	12.58	0.37
**Continues on the next page**					

[a]Unstandardised regression coefficient (β) and *P* value for maximal muscular strength in the model adjusted for age, education, smoking, use of alcohol, and diet (fruits and vegetables, meat, fish, and chicken). In the documentation of regression coefficient note that -7.638e-04 means -0.0007638 and in the documentation of *P* values note that *P*=8.894e-02 means *P*=0.08894 etc.
[b]R^2 is the coefficient of determination for the model with age, maximal muscular strength, education, smoking, use of alcohol, and diet (fruits and vegetables, meat, fish, and chicken) (independent variables) in relation to each metabolome measure (dependent variable).
[c]R^2 is the coefficient of determination for the model with age, education, smoking, use of alcohol, and diet (fruits and vegetables, meat, fish, and chicken) as independent variables.
[d]ΔR^2 is the difference of the coefficients of determination between the models [(age, maximal muscular strength, education, smoking, use of alcohol, and diet (fruits and vegetables, meat, fish, and chicken)) – (age, education, smoking, use of alcohol, and diet (fruits and vegetables, meat, fish, and chicken))] = additional explanation rate of muscular strength after age, education, smoking, use of alcohol, and diet (fruits and vegetables, meat, fish, and chicken) in the model.

© 2019 Kujala UM et al. *JAMA Network Open.*

eTable 8 continues from the previous page

Metabolome measure	β[a]	*P*[a]	R^{2b} (%)	R^{2c} (%)	ΔR^{2d} (%)
**Lipoprotein particle concentration**					
**Fatty acids**					
Total fatty acids	1.094e-04	0.1712	14.36	14.07	0.29
Unsaturation degree	1.721e-05	0.3802	9.17	9.05	0.13
Omega-3 FA	1.267e-04	1.193e-03	16.90	15.32	1.57
Omega-3 FA ratio	8.328e-04	3.877e-04	15.09	13.16	1.93
Docosahexaenoic acid	3.5485e-05	4.076e-03	16.38	15.13	1.24
Polyunsaturated FA	3.870e-04	0.1286	13.39	13.04	0.36
Omega-6 FA	2.602e-04	0.2377	12.54	12.33	0.22
Omega-6 FA ratio	-0.001858	7.903e-02	8.66	8.15	0.51
Linoleic acid	1.364e-04	0.4821	11.14	11.06	0.08
Saturated FA	1.255e-04	0.1281	14.14	13.78	0.36
Saturated FA ratio	6.352e-04	0.2467	2.17	1.93	0.23
Monounsaturated FA	1.259e-04	0.2289	13.78	13.56	0.22
**Metabolic substrates**					
Glucose	3.533e-04	9.756e-03	8.81	7.72	1.09
Glycerol	-2.227e-04	8.733e-02	4.52	4.03	0.50
Acetoacetate	-0.001051	9.177e-05	6.99	4.41	2.57
3-hydroxybuturate	-8.056e-04	2.272e-04	8.69	6.45	2.24
Acetate	-3.235e-05	0.7239	1.97	1.95	0.02
Citrate	-1.140e-05	2.258e-02	3.72	2.83	0.90
Lactate	-7.867e-05	0.4812	2.64	2.55	0.09
Pyruvate	-1.288e-04	0.1265	3.58	3.18	0.41
**Amino acids**					
Isoleucine (BCAA)	1.551e-04	8.471e-02	1.42	0.90	0.52
Leucine (BCAA)	1.431e-04	3.384e-02	2.82	2.04	0.78
Valine (BCAA)	2.277e-05	2.636e-02	3.92	3.07	0.85
Alanine	3.922e-05	4.648e-02	3.84	3.16	0.68
Glutamine	-7.215e-05	1.384e-02	18.69	17.80	0.89
Glycine	-9.590e-05	6.009e-02	1.73	1.11	0.62
Histidine	1.512e-06	0.5799	2.81	2.76	0.05
Phenylalanine	1.650e-04	5.407e-04	6.08	4.06	2.02
Tyrosine	1.761e-05	1.469e-06	5.93	1.97	3.97
**Miscellaneous**					
Glycoproteins	5.553e-05	0.3125	6.38	6.21	0.17
Creatinine	1.627e-05	3.169e-06	8.92	5.33	3.59
Albumine	-2.600e-06	0.1330	23.16	22.85	0.31

[a]Unstandardised regression coefficient (β) and *P* value for maximal muscular strength in the model adjusted for age, education, smoking, use of alcohol, and diet (fruits and vegetables, meat, fish, and chicken). In the documentation of regression coefficient note that -7.638e-04 means -0.0007638 and in the documentation of *P* values note that *P*=8.894e-02 means *P*=0.08894 etc.

[b]R^2 is the coefficient of determination for the model with age, maximal muscular strength, education, smoking, use of alcohol, and diet (fruits and vegetables, meat, fish, and chicken) (independent variables) in relation to each metabolome measure (dependent variable).

[c]R^2 is the coefficient of determination for the model with age, education, smoking, use of alcohol, and diet (fruits and vegetables, meat, fish, and chicken) as independent variables.

[d]ΔR^2 is the difference of the coefficients of determination between the models [(age, maximal muscular strength, education, smoking, use of alcohol, and diet (fruits and vegetables, meat, fish, and chicken)) – (age, education, smoking, use of alcohol, and diet (fruits and vegetables, meat, fish, and chicken))] = additional explanation rate of muscular strength after age, education, smoking, use of alcohol, and diet (fruits and vegetables, meat, fish, and chicken) in the model.

© 2019 Kujala UM et al. *JAMA Network Open.*

eTable 9. Linear Regression Analysis of the Age-Adjusted and Physical Activity–Adjusted Association of Aerobic Fitness With Serum Metabolome Measures

Metabolome measure	β[a]	*P*[a]	R^2[b] (%)	R^2[c] (%)	ΔR^2[d] (%)
**Lipoprotein particle concentration**					
Extremely large VLDL	-5.170e-02	3.858e-09	8.60	2.88	5.72
Very large VLDL	-8.829e-02	4.298e-09	8.04	2.32	5.72
Large VLDL	-4.713e-02	6.884e-12	10.33	2.63	7.70
Medium VLDL	-2.025e-02	2.089e-16	15.92	5.37	10.54
Small VLDL	-1.326e-02	1.154e-17	21.39	10.65	10.75
Very small VLDL	-2.365e-10	1.662e-11	24.76	18.55	6.21
IDL	-4.968e-10	3.132e-06	17.93	14.74	3.18
Large LDL	-8.849e-10	3.303e-06	17.39	14.20	3.19
Medium LDL	-8.125e-10	9.059e-07	18.17	14.65	3.53
Small LDL	-8.826e-10	1.608e-06	18.15	14.79	3.36
Very large HDL	5.524e-09	1.905e-16	12.82	1.85	10.96
Large HDL	1.753e-08	2.608e-26	22.50	5.61	16.88
Medium HDL	6.146e-09	1.054e-06	9.60	5.75	3.85
Small HDL	-7.079e-09	1.223e-04	4.47	1.97	2.50
**Lipoprotein particle size**					
VLDL diameter	-0.04191	4.678e-12	9.66	1.77	7.88
LDL diameter	1.561e-03	3.324e-02	2.14	1.36	0.78
HDL diameter	0.01089	1.584e-26	21.97	4.80	17.16
**Apolipoproteins**					
ApoA1	4.026e-03	5.726e-10	7.54	1.11	6.43
ApoB	-6.037e-03	2.814e-14	23.16	14.97	8.19
ApoB / ApoA1	-6.402e-03	2.354e-25	29.34	14.60	14.74
**Triglycerides**					
Total triglycerides	-1.574e-02	2.607e-18	19.05	7.51	11.54
VLDL-TG	-2.036e-02	6.683e-18	16.78	5.22	11.56
IDL-TG	-7.380e-03	3.072e-12	21.90	14.96	6.94
LDL-TG	-9.420e-04	1.405e-09	24.62	19.63	4.99
HDL-TG	-4.975e-03	2.635e-06	11.14	7.69	3.50
**Cholesterol**					
Total cholesterol	-0.009692	7.213e-03	13.54	12.44	1.10
VLDL-C	-1.280e-02	3.569e-14	21.02	12.68	8.34
IDL-C	-3.200e-03	2.293e-05	15.83	13.15	2.68
LDL-C	-0.008814	1.925e-05	16.10	13.38	2.72
HDL-C	1.010e-02	4.281e-19	15.60	2.97	12.64
HDL_2-C	9.755e-03	1.015e-20	17.84	4.31	13.53
HDL_3-C	3.057e-04	2.001e-02	8.20	7.33	0.87
Esterified cholesterol	-0.006753	9.634e-03	12.96	11.94	1.03
Free cholesterol	-2.939e-03	3.538e-03	14.86	13.59	1.28
**Continues on the next page**					

[a]Unstandardised regression coefficient (β) and *P* value for aerobic fitness in the model adjusted for age, aerobic physical activity volume, and strength training volume. In the documentation of regression coefficient note that -5.170e-02 means -0.05170 and in the documentation of *P* values note that *P*=3.858e-09 means *P*=0.00000000358 etc.

[b]R^2 is the coefficient of determination for the model with age, aerobic fitness, aerobic physical activity volume, and strength training volume (independent variables) in relation to each metabolome measure (dependent variable).

[c]R^2 is the coefficient of determination for the model with age, aerobic physical activity volume, and strength training volume as independent variables.

[d]ΔR^2 is the difference of the coefficients of determination between the models [(age, aerobic fitness, aerobic physical activity volume, and strength training volume) – (age, aerobic physical activity volume, and strength training volume) = additional explanation rate of aerobic fitness after age, aerobic physical activity volume, and strength training volume in the model.

© 2019 Kujala UM et al. *JAMA Network Open.*

eTable 9 continues from the previous page

Metabolome measure	β[a]	*P*[a]	R^{2b} (%)	R^{2c} (%)	ΔR^{2d} (%)
**Fatty acids**					
Total fatty acids	-4.985e-03	3.825e-08	18.07	13.60	4.47
Unsaturation degree	1.778e-03	1.037e-15	16.37	6.37	10.01
Omega-3 FA	-1.014e-03	2.430e-02	17.00	16.26	0.74
Omega-3 FA ratio	0.009330	6.789e-04	12.17	10.37	1.80
Docosahexaenoic acid	-2.890e-04	4.354e-02	16.20	15.60	0.60
Polyunsaturated FA	-0.008956	2.072e-03	15.17	13.74	1.42
Omega-6 FA	-0.007943	1.596e-03	14.32	12.81	1.51
Omega-6 FA ratio	0.09626	5.572e-16	16.00	5.75	10.25
Linoleic acid	-0.006396	3.903e-03	12.70	11.41	1.29
Saturated FA	-5.369e-03	8.877e-09	18.29	13.41	4.89
Saturated FA ratio	-1.382e-02	2.762e-02	1.68	0.84	0.84
Monounsaturated FA	-8.438e-03	1.127e-12	18.61	11.05	7.56
**Metabolic substrates**					
Glucose	-3.015e-03	5.638e-02	6.19	5.59	0.60
Glycerol	-1.447e-02	4.291e-24	20.27	4.57	15.69
Acetoacetate	-1.130e-02	2.668e-04	5.38	3.15	2.23
3-hydroxybuturate	-1.030e-02	5.400e-05	6.44	3.73	2.71
Acetate	-1.925e-04	0.8565	0.36	0.36	0.01
Citrate	-2.164e-05	0.7103	1.33	1.31	0.02
Lactate	-4.028e-03	1.878e-03	2.67	1.01	1.66
Pyruvate	-4.073e-03	2.300e-05	5.68	2.63	3.04
**Amino acids**					
Isoleucine (BCAA)	-6.462e-03	2.933e-10	7.35	0.68	6.67
Leucine (BCAA)	-4.928e-03	1.269e-10	7.79	0.87	6.92
Valine	-4.020e-04	6.809e-04	3.86	1.90	1.96
Alanine	-7.967e-04	4.576e-04	4.13	2.05	2.08
Glutamine	1.810e-03	5.849e-08	21.74	17.57	4.17
Glycine	1.530e-03	7.969e-03	2.47	1.26	1.21
Histidine	-2.880e-05	0.3626	1.99	1.85	0.14
Phenylalanine	-3.813e-03	4.104e-12	8.32	0.28	8.05
Tyrosine	-2.097e-04	5.874e-07	6.18	2.00	4.19
**Miscellaneous**					
Glycoproteins	-6.801e-03	1.989e-28	20.39	1.39	19.00
Creatinine	4.863e-05	0.2339	4.28	4.05	0.24
Albumine	3.307e-05	9.882e-02	21.22	20.84	0.38

[a]Unstandardised regression coefficient (β) and *P* value for aerobic fitness in the model adjusted for age, aerobic physical activity volume, and strength training volume. In the documentation of regression coefficient note that -5.170e-02 means -0.05170 and in the documentation of *P* values note that *P*=3.858e-09 means *P*=0.00000000358 etc.
[b]R^2 is the coefficient of determination for the model with age, aerobic fitness, aerobic physical activity volume, and strength training volume (independent variables) in relation to each metabolome measure (dependent variable).
[c]R^2 is the coefficient of determination for the model with age, aerobic physical activity volume, and strength training volume as independent variables.
[d]ΔR^2 is the difference of the coefficients of determination between the models [(age, aerobic fitness, aerobic physical activity volume, and strength training volume) – (age, aerobic physical activity volume, and strength training volume) = additional explanation rate of aerobic fitness after age, aerobic physical activity volume, and strength training volume in the model.

© 2019 Kujala UM et al. *JAMA Network Open.*

eTable 10. Linear Regression Analysis of the Age-Adjusted and Physical Activity–Adjusted Association of Maximal Muscular Strength With Serum Metabolome Measures

Metabolome measure	β[a]	*P*[a]	R^{2b} (%)	R^{2c} (%)	ΔR^{2d} (%)
**Lipoprotein particle concentration**					
Extremely large VLDL	6.317e-04	0.4071	2.99	2.87	0.12
Very large VLDL	1.277e-04	0.9221	2.32	2.32	0.00
Large VLDL	4.504e-05	0.9401	2.63	2.63	0.00
Medium VLDL	2.585e-04	0.2333	5.61	5.37	0.24
Small VLDL	2.156e-04	0.1144	11.04	10.65	0.39
Very small VLDL	5.916e-12	5.317e-02	19.08	18.55	0.53
IDL	1.126e-11	0.2206	14.97	14.74	0.23
Large LDL	2.087e-11	0.2037	14.45	14.20	0.25
Medium LDL	1.770e-11	0.2156	14.87	14.65	0.22
Small LDL	1.820e-11	0.2518	14.99	14.79	0.20
Very large HDL	-3.933e-11	0.5060	1.93	1.85	0.08
Large HDL	-2.788e-10	5.972e-02	6.20	5.61	0.59
Medium HDL	-3.159e-10	3.573e-03	7.14	5.75	1.39
Small HDL	-2.883e-10	6.882e-02	2.54	1.97	0.57
**Lipoprotein particle size**					
VLDL diameter	3.065e-04	0.5625	1.83	1.77	0.06
LDL diameter	-1.160e-04	6.445e-02	1.95	1.36	0.59
HDL diameter	-1.205e-04	0.1886	5.09	4.80	0.29
**Apolipoproteins**					
ApoA1	-9.258e-05	0.1009	1.58	1.11	0.47
ApoB	1.490e-04	3.194e-02	15.65	14.97	0.68
ApoB / ApoA1	1.521e-04	5.641e-03	15.74	14.60	1.14
**Triglycerides**					
Total triglycerides	2.066e-04	0.1947	7.78	7.51	0.27
VLDL-TG	2.229e-04	0.2850	5.41	5.22	0.19
IDL-TG	8.237e-05	0.3728	15.08	14.96	0.12
LDL-TG	1.493e-05	0.2693	19.80	19.63	0.17
HDL-TG	1.197e-04	0.1905	8.00	7.69	0.31
**Cholesterol**					
Total cholesterol	2.634e-04	0.3947	12.55	12.44	0.11
VLDL-C	2.915e-04	4.877e-02	13.27	12.68	0.59
IDL-C	8.468e-05	0.1934	13.41	13.15	0.26
LDL-C	2.055e-04	0.2476	13.58	13.38	0.20
HDL-C	-2.331e-04	1.957e-02	3.89	2.97	0.92
HDL_2-C	-2.107e-04	2.292e-02	5.17	4.31	0.86
HDL_3-C	-1.690e-05	0.1333	7.70	7.33	0.37
Esterified cholesterol	1.659e-04	0.4583	12.02	11.94	0.08
Free cholesterol	9.782e-05	0.2578	13.78	13.59	0.19
**Continues on the next page**					

[a]Unstandardised regression coefficient (β) and *P* value for muscle strength in the model adjusted for age, aerobic physical activity volume, and strength training volume. In the documentation of regression coefficient note that 6.317e-04 means 0.0006317 and in the documentation of *P* values note that *P*=5.317e-02 means *P*=0.05317 etc.

[b]R^2 is the total coefficient of determination for the model with age, maximal muscular strength, aerobic physical activity volume, and strength training volume (independent variables) in relation to each metabolome measure (dependent variable).

[c]R^2 is the total coefficient of variation for the model with age, aerobic physical activity volume, and strength training volume as independent variables.

[d]ΔR^2 is the difference of the coefficients of determination between the models [(age, maximal muscular strength, aerobic physical activity volume, and strength training volume) – (age, aerobic physical activity volume, and strength training volume) = additional explanation rate of maximal muscular strength after age, aerobic physical activity volume, and strength training volume in the model.

© 2019 Kujala UM et al. *JAMA Network Open.*

eTable 10 continues from the previous page

Metabolome measure	β[a]	*P*[a]	R^{2b} (%)	R^{2c} (%)	ΔR^{2d} (%)
**Fatty acids**					
Total fatty acids	7.778e-05	0.3245	13.75	13.60	0.15
Unsaturation degree	1.595e-05	0.4153	6.48	6.37	0.11
Omega-3 FA	1.071e-04	5.595e-03	17.38	16.26	1.12
Omega-3 FA ratio	8.052e-04	6.468e-04	12.18	10.37	1.81
Docosahexaennoic acid	2.960e-05	1.609e-02	16.46	15.60	0.86
Polyunsaturated FA	2.709e-04	0.2803	13.92	13.74	0.18
Omega-6 FA	1.637e-04	0.4510	12.90	12.81	0.09
Omega-6 FA ratio	-0.0015164	0.1489	6.10	5.75	0.35
Linoleic acid	5.614e-05	0.7691	11.43	11.41	0.02
Saturated FA	1.064e-04	0.1909	13.67	13.41	0.26
Saturated FA ratio	0.001067	4.800e-02	1.52	0.84	0.68
Monounsaturated FA	6.309e-05	0.5449	11.11	11.05	0.06
**Metabolic substrates**					
Glucose	3.652e-04	6.805e-03	6.80	5.59	1.21
Glycerol	-2.158e-04	9.110e-02	5.05	4.57	0.48
Acetoacetate	-9.521e-04	3.498e-04	5.30	3.15	2.15
3-hydroxybuturate	-7.377e-04	6.914e-04	5.65	3.73	1.92
Acetate	-8.530e-06	0.9253	0.36	0.36	0.00
Citrate	-6.363e-06	0.2014	1.59	1.31	0.28
Lactate	-1.001e-04	0.3685	1.15	1.01	0.14
Pyruvate	-1.521e-04	6.656e-02	3.21	2.63	0.58
**Amino acids**					
Isoleucine (BCAA)	1.921e-04	3.104e-02	1.49	0.68	0.81
Leucine (BCAA)	1.665e-04	1.237e-02	1.95	0.87	1.08
Valini (BCAA)	2.743e-05	6.830e-03	3.15	1.90	1.25
Alanine	3.610e-05	6.444e-02	2.64	2.05	0.59
Glutamine	-7.475e-05	9.540e-03	18.55	17.57	0.98
Glycine	-1.113e-04	2.424e-02	2.14	1.26	0.88
Histidine	2.714e-06	0.3160	2.02	1.85	0.29
Phenylalanine	1.464e-04	2.221e-03	1.89	0.28	1.61
Tyrosine	1.819e-05	4.0413e-07	6.30	1.20	5.10
**Miscellaneous**					
Glycoproteins	3.487e-05	0.5293	1.46	1.39	0.07
Creatinine	1.734e-05	5.287e-07	8.18	4.05	4.13
Albumin	-2.070e-06	0.2273	21.04	20.84	0.20

[a]Unstandardised regression coefficient (β) and *P* value for muscle strength in the model adjusted for age, aerobic physical activity volume, and strength training volume. In the documentation of regression coefficient note that 6.317e-04 means 0.0006317 and in the documentation of *P* values note that *P*=5.317e-02 means *P*=0.05317 etc.

[b]R^2 is the total coefficient of determination for the model with age, maximal muscular strength, aerobic physical activity volume, and strength training volume (independent variables) in relation to each metabolome measure (dependent variable).

[c]R^2 is the total coefficient of variation for the model with age, aerobic physical activity volume, and strength training volume as independent variables.

[d]ΔR^2 is the difference of the coefficients of determination between the models [(age, maximal muscular strength, aerobic physical activity volume, and strength training volume) – (age, aerobic physical activity volume, and strength training volume) = additional explanation rate of maximal muscular strength after age, aerobic physical activity volume, and strength training volume in the model.

© 2019 Kujala UM et al. *JAMA Network Open.*

eTable 11. Linear Regression Analysis of the Age-Adjusted And Body Fat Percentage–Adjusted Association of Aerobic Fitness With Serum Metabolome Measures

Metabolome measure	β[a]	*P*[a]	R^{2b} (%)	R^{2c} (%)	ΔR^{2d} (%)
**Lipoprotein particle concentration**					
Extremely large VLDL	-0.02876	1.223e-02	9.98	8.98	1.00
Very large VLDL	-4.165e-02	3.281e-02	10.36	9.63	0.73
Large VLDL	-0.02417	6.505e-03	12.56	11.42	1.14
Medium VLDL	-1.033e-02	1.043e-03	18.68	17.32	1.36
Small VLDL	-5.693e-03	3.636e-03	25.21	24.08	1.13
Very small VLDL	-5.895e-11	0.1876	27.87	27.65	0.22
IDL	-5.639e-11	0.6838	19.18	19.15	0.03
Large LDL	-8.642e-11	0.7262	18.76	18.74	0.02
Medium LDL	-9.190e-11	0.6674	19.86	19.83	0.03
Small LDL	-8.082e-11	0.7339	19.96	19.94	0.02
Very large HDL	3.557e-09	4.786e-05	13.10	10.54	2.56
Large HDL	1.155e-08	4.143e-08	23.76	19.61	4.15
Medium HDL	5.684e-09	6.525e-04	9.56	7.69	1.87
Small HDL	-6.020e-10	0.8032	6.83	6.82	0.01
**Lipoprotein particle size**					
VLDL diameter	-2.562e-02	1.237e-03	10.67	9.01	1.66
LDL diameter	4.101e-04	0.6753	2.36	2.33	0.03
HDL diameter	0.006512	4.479e-07	24.13	20.65	3.48
**Apolipoproteins**					
ApoA1	3.799e-03	1.085e-05	6.30	3.05	3.25
ApoB	-0.002006	4.467e-02	26.73	26.21	0.52
ApoB / ApoA1	-0.003170	3.126e-05	33.24	31.17	2.07
**Triglycerides**					
Total triglycerides	-0.007524	1.009e-03	22.46	20.97	1.49
VLDL-TG	-0.01046	5.124e-04	19.75	18.03	1.72
IDL-TG	-2.308e-03	8.849e-02	25.08	24.70	0.38
LDL-TG	-1.696e-04	0.3963	27.62	27.53	0.09
HDL-TG	-1.221e-03	0.3798	13.00	12.88	0.12
**Cholesterol**					
Total cholesterol	0.003536	0.4551	14.11	14.02	0.09
VLDL-C	-0.00487	2.342e-02	24.41	23.72	0.69
IDL-C	-2.838e-04	0.7733	16.66	16.65	0.01
LDL-C	-0.0005664	0.8329	17.12	17.11	0.01
HDL-C	0.007383	5.327e-07	15.65	11.83	3.82
HDL_2-C	0.006856	4.042e-07	18.25	14.47	3.78
HDL_3-C	4.862e-04	5.758e-03	7.78	6.53	1.25
Esterified cholesterol	0.002654	0.4380	13.50	13.41	0.09
Free cholesterol	8.796e-04	0.5058	15.47	15.40	0.07
**Continues on the next page**					

[a]Unstandardised regression coefficient (β) and *P* value for aerobic fitness in the model adjusted for age and body fat percentage. In the documentation of regression coefficient note that -4.165e-02 means -0.04165 and in the documentation of *P* values note that *P*=1.223e-02 means *P*=0.01223 etc.

[b]R^2 is the total coefficient of determination for the model with age, aerobic fitness, and fat percentage (independent variables) in relation to each metabolome measure (dependent variable).

[c]R^2 is the total coefficient of determination for the model with age and fat percentage in the model as independent variables.

[d]ΔR^2 is the difference of the coefficients of determination between the models [(age, aerobic fitness, and fat percentage) – (age, fat percentage)] = additional explanation rate of aerobic fitness after age and fat percentage in the model.

© 2019 Kujala UM et al. *JAMA Network Open.*

eTable 11 continues from the previous page

Metabolome measure	β[a]	*P*[a]	R^{2b} (%)	R^{2c} (%)	ΔR^{2d} (%)
**Fatty acids**					
Total fatty acids_ln	-5.735e-04	0.6212	21.57	21.53	0.04
Unsaturation degree	0.001210	3.588e-05	14.67	12.05	2.62
Omega-3 FA	9.447e-04	0.1099	17.45	17.08	0.37
Omega-3 FA ratio	0.01343	3.475e-04	7.39	5.27	2.12
Docosahexaenoic acid	2.594e-04	0.1699	16.07	15.79	0.28
Polyunsaturated FA	0.003330	0.3799	16.64	16.53	0.11
Omega-6 FA	0.002384	0.4677	15.86	15.78	0.08
Omega-6 FA ratio	0.04787	1.766e-03	18.41	16.99	1.42
Linoleic acid	0.002246	0.4391	14.04	13.95	0.09
Saturated FA	-0.001001	0.4022	21.48	21.39	0.09
Saturated FA ratio	-0.01514	7.180e-02	1.02	0.45	0.57
Monounsaturated FA	-0.002287	0.1277	23.21	22.90	0.31
**Metabolic substrates**					
Glucose	-6.073e-04	0.7731	6.18	6.16	0.02
Glycerol	-0.008664	1.742e-06	22.90	19.73	3.17
Acetoacetate	-0.01951	3.078e-06	4.15	0.39	3.76
3-hydroxybuturate	-0.01343	9.167e-05	3.92	1.29	2.63
Acetate	4.647e-04	0.7450	0.32	0.30	0.02
Citrate	5.286e-05	0.4997	0.74	0.66	0.08
Lactate	0.001540	0.3605	5.82	5.68	0.14
Pyruvate	-0.002649	3.896e-02	4.97	4.24	0.73
**Amino acids**					
Isoleucine (BCAA)	-0.001263	0.3346	12.25	12.10	0.15
Leucine (BCAA)	-7.088e-04	0.4683	13.48	13.40	0.08
Valine	9.948e-05	0.5174	7.78	7.72	0.06
Alanine	3.785e-04	0.1993	7.71	7.44	0.27
Glutamine	9.149e-04	3.777e-02	22.68	22.09	0.59
Glycine	3.646e-04	0.6382	2.77	2.72	0.05
Histidine	1.810e-05	0.6669	1.60	1.57	0.03
Phenylalanine	0.0005524	0.4128	18.96	18.87	0.09
Tyrosine	5.704e-05	0.2958	9.16	8.98	0.18
**Miscellaneous**					
Glycoproteins	-0.003443	5.614e-06	25.34	22.58	2.76
Creatinine	6.959e-05	0.2102	1.45	1.18	0.27
Albumine	2.212e-05	0.4121	20.59	20.49	0.10

[a]Unstandardised regression coefficient (β) and *P* value for aerobic fitness in the model adjusted for age and body fat percentage. In the documentation of regression coefficient note that -4.165e-02 means -0.04165 and in the documentation of *P* values note that *P*=1.223e-02 means *P*=0.01223 etc.
[b]R^2 is the total coefficient of determination for the model with age, aerobic fitness, and fat percentage (independent variables) in relation to each metabolome measure (dependent variable).
[c]R^2 is the total coefficient of determination for the model with age and fat percentage in the model as independent variables.
[d]ΔR^2 is the difference of the coefficients of determination between the models [(age, aerobic fitness, and fat percentage) – (age, fat percentage)] = additional explanation rate of aerobic fitness after age and fat percentage in the model.

© 2019 Kujala UM et al. *JAMA Network Open.*

eTable 12. Linear Regression Analysis of the Age-Adjusted And Body Fat Percentage–Adjusted Association of Maximal Muscular Strength With Serum Metabolome Measures

Metabolome measure	β[a]	*P*[a]	R^{2b} (%)	R^{2c} (%)	$ΔR^{2d}$ (%)
**Lipoprotein particle concentration**					
Extremely large VLDL	8.492e-05	0.9049	8.98	8.98	0.00
Very large VLDL	-7.56109e-04	0.5308	9.70	9.63	0.07
Large VLDL	-4.933e-04	0.3695	11.54	11.42	0.12
Medium VLDL	6.217e-05	0.7505	17.14	17.13	0.01
Small VLDL	1.002e-04	0.4084	24.17	24.08	0.09
Very small VLDL	4.748e-12	8.529e-02	28.03	27.65	0.38
IDL	9.897e-12	0.2465	19.35	19.15	0.20
Large LDL	1.883e-11	0.2160	18.96	18.74	0.22
Medium LDL	1.596e-11	0.2263	20.04	19.83	0.21
Small LDL	1.626e-11	0.2675	20.11	19.94	0.17
Very large HDL	2.050e-11	0.7063	10.56	10.54	0.02
Large HDL	-1.124e-10	0.3932	19.72	19.61	0.11
Medium HDL	-2.873e-10	5.314e-03	8.95	7.69	1.26
Small HDL	-3.931e-10	8.167e-03	7.96	6.82	1.14
**Lipoprotein particle size**					
VLDL diameter	-1.894e-04	0.7002	9.04	9.01	0.03
LDL diameter	-9.534e-05	0.1142	2.76	2.33	0.43
HDL diameter	-1.417e-05	0.8603	20.65	20.65	0.00
**Apolipoproteins**					
ApoA1	-5.287e-05	0.3250	3.22	3.05	0.17
ApoB	1.072e-04	8.226e-02	26.60	26.21	0.39
ApoB / ApoA1	1.031e-04	2.909e-02	31.74	31.17	0.57
**Triglycerides**					
Total triglycerides	5.560e-05	0.6952	20.99	20.97	0.02
VLDL-TG	2.168e-05	0.9076	18.03	18.03	0.00
IDL-TG	4.165e-05	0.6188	24.73	24.70	0.03
LDL-TG	1.018e-05	0.4093	27.62	27.53	0.09
HDL-TG	7.506e-05	0.3818	13.00	12.88	0.12
**Cholesterol**					
Total cholesterol	2.789e-04	0.3395	14.16	14.02	0.14
VLDL-C	1.875e-04	0.1584	23.99	23.72	0.27
IDL-C	7.675e-05	0.2064	16.88	16.65	0.23
LDL-C	1.979e-04	0.2319	17.32	17.11	0.21
HDL-C	-1.394e-04	0.1288	12.19	11.83	0.36
HDL_2-C	-1.236e-04	0.1430	14.79	14.47	0.32
HDL_3-C	-1.118044e-05	0.3049	6.70	6.53	0.17
Esterified cholesterol	1.773e-04	0.40136	13.52	13.41	0.11
Free cholesterol	1.020e-04	0.2108	15.63	15.40	0.23
**Continues on the next page**					

[a]Unstandardised regression coefficient (β) and *P* value for maximal muscular strength in the model adjusted for age and body fat percentage. In the documentation of regression coefficient note that 8.492e-05 means 0.00008492 and in the documentation of *P* values note that *P*=8.529e-02 means *P*=0.08529 etc.

[b]R^2 is the total coefficient of determination for the model with age, maximal muscular strength and fat percentage (independent variables) in relation to each metabolome measure (dependent variable).

[c]R^2 is the total coefficient of determination for the model with age and fat percentage as independent variables.

[d]$ΔR^2$ is the difference in the coefficients of determination between the models [(age, maximal muscular strength, and fat percentage) – (age and fat percentage)] = additional explanation rate of maximal muscular strength after age and fat percentage in the model.

© 2019 Kujala UM et al. *JAMA Network Open.*

eTable 12 continues from the previous page

**Metabolome measure**	**β[a]**	***P*[a]**	**R^{2b} (%)**	**R^{2c} (%)**	**ΔR^{2d} (%)**
**Fatty acids**					
Total fatty acids	4.652e-05	0.5178	21.59	21.53	0.06
Unsaturation degree	4.421e-05	1.533e-02	12.96	12.05	0.91
Omega-3 FA	1.206e-04	9.493e-04	18.66	17.08	1.58
Omege-3 FA ratio	0.001081	3.174e-06	8.84	5.27	3.57
Docosahexaenoic acid	3.379e-05	3.830e-03	17.02	15.79	1.23
Polyunsaturated FA	2.965e-04	0.2071	16.76	16.53	0.23
Omega-6 FA	1.758e-04	0.3876	15.89	15.78	0.11
Omega-6 FA ratio	-3.453e-04	0.7171	17.01	16.99	0.02
Linoleic acid	7.200e-05	0.6891	13.97	13.95	0.02
Saturated FA	6.433e-05	0.3851	21.49	21.39	0.10
Saturated FA ratio	6.851e-04	0.1893	0.76	0.45	0.31
Monounsaturated FA	-7.555e-06	0.9354	22.90	22.90	0.00
**Metabolic substrates**					
Glucose	2.654e-04	4.081e-02	6.85	6.16	0.69
Glycerol	-3.969e-04	4.173e-04	21.47	19.73	1.74
Acetoacetate	-0.001175	5.860e-06	3.94	0.39	3.55
3-hydroxybuturate	-9.608e-04	5.486e-06	4.82	1.29	3.53
Acetate	-4.831e-06	0.9563	0.30	0.30	0.00
Citrate	-1.003e-05	3.764-02	1.42	0.66	0.76
Lactate	-9.995e-05	0.3360	5.83	5.68	0.15
Pyruvate	-2.032e-04	1.003e-02	5.37	4.24	0.13
**Amino acids**					
Isoleucine	1.203e-04	0.1361	12.45	12.10	0.35
Leucine	1.145e-04	5.726e-02	13.95	13.40	0.55
Valine	2.470e-05	9.023e-03	8.82	7.72	1.10
Alanine	3.451e-05	5.772e-02	8.03	7.44	0.59
Glutamine	-5.044e-05	6.304e-02	22.56	22.09	0.47
Glycine	-9.336e-05	5.070e-02	3.38	2.73	0.65
Histidine	1.149e-06	0.6579	1.60	1.57	0.03
Phenylalanine	1.444e-04	4.921e-04	20.59	18.87	1.72
Tyrosine	1.831e-05	3.650e-08	13.72	8.98	4.74
**Miscellaneous**					
Glycoproteins	-1.610e-05	0.7331	22.60	22.58	0.02
Creatinine	1.996e-05	3.419e-09	7.08	1.18	5.90
Albumine	-3.146e-06	5.837e-02	20.99	20.49	0.50

[a]Unstandardised regression coefficient (β) and *P* value for maximal muscular strength in the model adjusted for age and body fat percentage. In the documentation of regression coefficient note that 8.492e-05 means 0.00008492 and in the documentation of *P* values note that *P*=8.529e-02 means *P*=0.08529 etc.
[b]R^2 is the total coefficient of determination for the model with age, maximal muscular strength and fat percentage (independent variables) in relation to each metabolome measure (dependent variable).
[c]R^2 is the total coefficient of determination for the model with age and fat percentage as independent variables.
[d]ΔR^2 is the difference in the coefficients of determination between the models [(age, maximal muscular strength, and fat percentage) – (age and fat percentage)] = additional explanation rate of maximal muscular strength after age and fat percentage in the model.

© 2019 Kujala UM et al. *JAMA Network Open.*

eTable 13. Summary of the ΔR2 Values From Regression Models Indicating the Additional Variation Accounted for by Each Fitness Variable

	ΔR^2 (%) Between the models[a]							
	**(Age + AF) – Age**	**(Covariates[b] + AF) – Covariates[b]**	**(Age + PA[c] + AF) – (Age + PA[c])**	**(Age + Fat% + AF) – (Age + Fat%)**	**(Age + MS) – Age**	**(Covariates[b] + MS) – Covariates[b]**	**(Age + PA[c] + MS) – (Age + PA[c])**	**(Age + Fat% + MS) – (Age + Fat%)**
**Lipoprotein particle concentration**								
Extremely large VLDL	5.94[a] (-)	4.73[a] (-)	5.72[a] (-)	1.00 (-)	0.01 (+)	0.17 (+)	0.12 (+)	0.00
Very large VLDL	6.14[a] (-)	4.80[a] (-)	5.72[a] (-)	0.73 (-)	0.04 (-)	0.04 (+)	0.00	0.07 (-)
Large VLDL	8.34[a] (-)	6.28[a] (-)	7.70[a] (-)	1.14 (-)	0.06 (-)	0.06 (+)	0.00	0.12 (-)
Medium VLDL	11.09[a] (-)	8.78[a] (-)	10.54[a] (-)	1.36[a] (-)	0.05 (+)	0.47 (+)	0.24 (+)	0.01 (+)
Small VLDL	11.16[a] (-)	9.00[a] (-)	10.75[a] (-)	1.13 (-)	0.17 (+)	0.74 (+)	0.39 (+)	0.09 (+)
Very small VLDL	6.28[a] (-)	6.13[a] (-)	6.21[a] (-)	0.22 (-)	0.56 (+)	0.89 (+)	0.53 (+)	0.38 (+)
IDL	3.30[a] (-)	3.67[a] (-)	3.18[a] (-)	0.03 (-)	0.33 (+)	0.38 (+)	0.23 (+)	0.20 (+)
Large LDL	3.29[a] (-)	3.58[a] (-)	3.19[a] (-)	0.02 (-)	0.36 (+)	0.43 (+)	0.25 (+)	0.22 (+)
Medium LDL	3.60[a] (-)	3.76[a] (-)	3.53[a] (-)	0.03 (-)	0.34 (+)	0.45 (+)	0.22 (+)	0.21 (+)
Small LDL	3.44[a] (+)	3.55[a] (-)	3.36[a] (-)	0.02 (-)	0.30 (+)	0.41 (+)	0.20 (+)	0.17 (+)
Very large HDL	11.44[a] (+)	8.43[a] (+)	10.96[a] (+)	2.56[a] (+)	0.00	0.23 (-)	0.08 (-)	0.02 (+)
Large HDL	17.26[a] (+)	14.97[a] (+)	16.88[a] (+)	4.15[a] (+)	0.19 (-)	0.72 (-)	0.59 (-)	0.11 (-)
Medium HDL	3.47[a] (-)	4.45[a] (+)	3.85[a] (+)	1.87[a] (+)	1.35 (-)	0.97 (-)	1.39 (-)	1.26 (-)
Small HDL	2.96[a] (-)	1.23 (-)	2.50[a] (-)	0.01 (-)	1.04 (-)	0.13 (-)	0.57 (-)	1.14 (-)
**Lipoprotein particle size**								
VLDL diameter	8.45[a] (-)	6.27[a] (-)	7.88[a] (-)	1.66[a] (-)	0.01 (-)	0.13 (+)	0.06 (+)	0.03 (-)
LDL diameter	0.75 (+)	0.51 (+)	0.78 (+)	0.03 (+)	0.47 (-)	0.60 (-)	0.59 (-)	0.43 (-)
HDL diameter	17.85[a] (+)	14.45[a] (+)	17.16[a] (+)	3.48[a] (+)	0.03 (-)	0.50 (-)	0.29 (-)	0.00
**Apolipoproteins**								
ApoA1	6.27[a] (+)	3.81[a] (+)	6.43[a] (+)	3.25[a] (+)	0.18 (-)	0.36 (-)	0.47 (-)	0.17 (-)
ApoB	8.52[a] (-)	8.26[a] (-)	8.18[a] (-)	0.52 (-)	0.59 (+)	0.90 (+)	0.68 (+)	0.39 (+)
ApoB / ApoA1 ratio	15.13[a] (-)	14.49[a] (-)	14.73[a] (-)	2.07[a] (-)	0.81 (+)	1.27 (+)	0.29 (+)	0.57 (+)
**Triglycerides**								
Total triglycerides	12.16[a] (-)	9.73[a] (-)	11.54[a] (-)	1.49[a] (-)	0.07 (+)	0.52 (+)	0.27 (+)	0.02 (+)
VLDL-TG	12.19[a] (-)	9.57[a] (-)	11.56[a] (-)	1.72[a] (-)	0.03 (+)	0.42 (+)	0.19 (+)	0.00
IDL-TG	7.17[a] (-)	6.22[a] (-)	6.94[a] (-)	0.38 (-)	0.09 (+)	0.34 (+)	0.12 (+)	0.03 (+)
LDL-TG	5.18[a] (-)	4.81[a] (-)	4.99[a] (-)	0.09 (-)	0.17 (+)	0.38 (+)	0.17 (+)	0.09 (+)
HDL-TG	3.64[a] (-)	2.99[a] (-)	3.50[a] (-)	0.12 (-)	0.16 (+)	0.44 (+)	0.31 (+)	0.12 (+)
**Cholesterol**								
Total cholesterol	1.16 (-)	1.35 (-)	1.10 (-)	0.09 (+)	0.24 (+)	0.25 (+)	0.11 (+)	0.14 (+)
VLDL-C	8.68[a] (-)	7.70[a] (-)	8.34[a] (-)	0.69 (-)	0.39 (+)	0.80 (+)	0.59 (+)	0.27 (+)
IDL-C	2.80[a] (-)	3.25[a] (-)	2.68[a] (-)	0.01 (-)	0.38 (+)	0.39 (+)	0.26 (+)	0.23 (+)
LDL-C	2.78[a] (-)	3.05[a] (-)	2.72[a] (-)	0.01 (-)	0.34 (+)	0.39 (+)	0.20 (+)	0.21 (+)
HDL-C	12.69[a] (+)	11.47[a] (+)	12.64[a] (+)	3.82[a] (+)	0.46 (-)	0.89 (-)	0.92 (-)	0.36 (-)
HDL_2-C	13.57[a] (+)	12.27[a] (+)	13.53[a] (+)	3.78[a] (+)	0.42 (-)	0.89 (-)	0.86 (-)	0.32 (-)
HDL_3-C	0.90 (+)	0.82 (+)	0.87 (+)	1.25 (+)	0.16 (-)	0.16 (-)	0.37 (-)	0.17 (-)
Esterified cholesterol	1.09 (-)	1.27 (-)	1.02 (-)	0.09 (+)	0.20 (+)	0.21 (+)	0.08 (+)	0.11 (+)
Free cholesterol	1.31 (-)	1.53[a] (-)	1.28 (-)	0.07 (+)	0.35 (+)	0.37 (+)	0.19 (+)	0.23 (+)
**Fatty acids**								
Total fatty acids	4.81[a] (-)	4.39[a] (-)	4.47[a] (-)	0.04 (-)	0.12 (+)	0.29 (+)	0.15 (+)	0.06 (+)
Unsaturation degree	11.51[a] (+)	8.22[a] (+)	10.01[a] (+)	2.62[a] (+)	0.79 (+)	0.13 (+)	0.11 (+)	0.91 (+)
**Continues on the next page**								

Abbreviations: ΔR^2, the difference between the coefficients of determination between the models; AF, aerobic fitness; MS, muscular strength; fat%, body fat percentage.
\+ and – values in parenthesis indicate the direction of the association between the fitness variable and the metabolome measure.
Results of the original separate models are shown in eTables 5-12 in this Supplement.
[a]Statistically significant ΔR^2 value with $P \le .002$.
[b]Covariates include age, education, smoking, use of alcohol and dietary factors (consumption of vegetables, fruits, fish, chicken, and meat).
[c]PA, physical activity including minutes of aerobic training and minutes of strength training per week.

© 2019 Kujala UM et al. *JAMA Network Open.*

eTable 13 continues from the previous page

	ΔR² (%) Between the Models[a]							
	(Age + AF) – Age	(Covariates[b] + AF) – Covariates[b]	(Age + PA[c] + AF) – (Age + PA[c])	(Age + Fat% + AF) – (Age + Fat%)	(Age + MS) – Age	(Covariates[b] + MS) – Covariates[b]	(Age + PA[c] + MS) – (Age + PA[c])	(Age + Fat% + MS) – (Age + Fat%)
**Fatty acids; continues from the previous page**								
Omega-3 FA	0.61 (-)	1.11 (-)	0.74 (-)	0.37 (+)	1.83[a] (+)	1.57[a] (+)	1.12 (+)	1.58[a] (+)
Omega-3 FA ratio	2.43[a] (+)	0.79 (+)	1.80[a] (+)	2.12[a] (+)	3.67[a] (+)	1.93[a] (+)	1.81[a] (+)	3.57[a] (+)
Docosahexaenoic acid	0.40 (-)	1.03 (-)	0.60 (-)	0.28 (+)	1.47[a] (+)	1.24 (+)	0.86 (+)	1.23 (+)
Polyunsaturated FA	1.43 (-)	1.67[a] (-)	1.42 (-)	0.11 (+)	0.35 (+)	0.36 (+)	0.18 (+)	0.23 (+)
Omega-6 FA	1.56[a] (-)	1.71[a] (-)	1.51[a] (-)	0.08 (+)	0.20 (+)	0.22 (+)	0.09 (+)	0.11 (+)
Omega-6 FA ratio	11.05[a] (+)	8.49[a] (+)	10.25[a] (+)	1.42[a] (+)	0.04 (-)	0.51 (-)	0.35 (-)	0.02 (+)
Linoleic acid	1.35 (-)	1.44 (-)	1.29 (-)	0.09 (+)	0.07 (+)	0.08 (+)	0.02 (+)	0.02 (+)
Saturated FA	5.32[a] (-)	5.01[a] (-)	4.89[a] (-)	0.09 (-)	0.19 (+)	0.36 (+)	0.26 (+)	0.10 (+)
Saturated FA ratio	1.02 (-)	1.40 (-)	0.84 (-)	0.57 (-)	0.32 (+)	0.23 (+)	0.68 (+)	0.31 (+)
Monounsaturated FA	8.17[a] (-)	6.41[a] (-)	7.56[a] (-)	0.31 (-)	0.01 (+)	0.22 (+)	0.06 (+)	0.00
**Metabolic substrates**								
Glucose	0.56 (-)	0.38 (-)	0.60 (-)	0.02 (-)	0.80 (+)	1.09 (+)	1.21 (+)	0.69 (+)
Glycerol	18.19[a] (-)	16.17[a] (-)	15.69[a] (-)	3.17[a] (-)	1.42 (+)	0.50 (-)	0.48 (-)	1.74[a] (-)
Acetoacetate	3.13[a] (-)	2.58[a] (-)	2.23[a] (-)	3.76[a] (-)	3.38[a] (-)	2.57[a] (-)	2.15[a] (-)	3.55[a] (-)
3-hydroxybuturate	3.80[a] (-)	3.02[a] (-)	2.71[a] (-)	2.63[a] (-)	3.29[a] (-)	2.24[a] (-)	1.92[a] (-)	3.53[a] (-)
Acetate	0.00 (-)	0.03 (-)	0.01 (-)	0.02 (+)	0.00	0.02 (-)	0.00	0.00
Citrate	0.10 (-)	0.20 (-)	0.02 (-)	0.08 (+)	0.66 (-)	0.90 (-)	0.28 (-)	0.76 (-)
Lactate	1.59 (-)	1.73[a] (-)	1.66[a] (-)	0.14 (+)	0.10 (-)	0.09 (-)	0.14 (-)	0.15 (-)
Pyruvate	3.94[a] (-)	2.79[a] (-)	3.04[a] (-)	0.73 (-)	0.87 (-)	0.41 (-)	0.58 (-)	0.13 (-)
**Amino acids**								
Isoleucine (BCAA)	6.81[a] (-)	6.95[a] (-)	6.67[a] (-)	0.15 (-)	0.45 (+)	0.52 (+)	0.81 (+)	0.35 (+)
Leucine (BCAA)	6.96[a] (-)	6.88[a] (-)	6.92[a] (-)	0.08 (-)	0.66 (+)	0.78 (+)	1.08 (+)	0.55 (+)
Valine (BCAA)	1.96[a] (-)	2.71[a] (-)	1.96[a] (-)	0.06 (+)	1.18 (+)	0.85 (+)	1.25 (+)	1.10 (+)
Alanine	1.68[a] (-)	1.88[a] (-)	2.08[a] (-)	0.27 (+)	0.71 (+)	0.68 (+)	0.59 (+)	0.59 (+)
Glutamine	4.31[a] (+)	4.92[a] (+)	4.17[a] (+)	0.59 (+)	0.61 (-)	0.89 (-)	0.98 (-)	0.47 (-)
Glycine	1.38 (+)	1.79[a] (+)	1.21 (-)	0.05 (+)	0.80 (-)	0.62 (-)	0.88 (-)	0.65 (-)
Histidine	0.15 (-)	0.05 (-)	0.14 (-)	0.03 (+)	0.04 (+)	0.05 (+)	0.29 (+)	0.03 (+)
Phenylalanine	7.27[a] (-)	6.46[a] (-)	8.05[a] (-)	0.09 (+)	1.80 (+)	2.02a (+)	1.61 (+)	1.72[a] (+)
Tyrosine	3.13[a] (-)	3.88[a] (-)	4.19[a] (-)	0.18 (+)	4.83[a] (+)	3.97[a] (+)	5.10[a] (+)	4.74[a] (+)
**Miscellaneous**								
Glycoproteins	19.65[a] (-)	15.90[a] (-)	19.00[a] (-)	2.76[a] (-)	0.01 (-)	0.17 (+)	0.07 (+)	0.02 (-)
Creatinine	0.53 (+)	0.03 (+)	0.23 (+)	0.27 (+)	5.74[a] (+)	3.59[a] (+)	4.13[a] (+)	5.90[a] (+)
Albumin	0.13 (+)	0.16 (+)	0.38 (+)	0.10 (+)	0.49 (-)	0.31 (-)	0.20 (-)	0.50 (-)

Abbreviations: ΔR², the difference between the coefficients of determination between the models; AF, aerobic fitness; MS, muscular strength; fat%, body fat percentage.
+ and – values in parenthesis indicate the direction of the association between the fitness variable and the metabolome measure.
Results of the original separate models are shown in eTables 5-12 in this Supplement.
[a]Statistically significant ΔR² value with *P*≤.002.
[b]Covariates include age, education, smoking, use of alcohol and dietary factors (consumption of vegetables, fruits, fish, chicken, and meat).
[c]PA, physical activity including minutes of aerobic training and minutes of strength training per week.

© 2019 Kujala UM et al. *JAMA Network Open.*

eTable 14. Additional Explanation Rate for Absolute and Relative-to-Body-Weight Maximal Muscular Strength After Adjusting for Age and After Adjusting for Age and Body Fat Percentage

Metabolome measure	R^2 (%) for age	R^2 (%) for age and absolute strength	Additional R^2 (%) for absolute strength after age	R^2 (%) for age and relative strength	Additional R^2 (%) for relative strength after age	R^2 (%) for age and fat%	R^2 (%) for age, fat% and absolute strength	Additional R^2 (%) for absolute strength after age and fat%	R^2 (%) for age, fat% and relative strength	Additional R^2 (%) for relative strength after age and fat%
**Lipoprotein particle concentration**										
Extremely large VLDL	2.30[a] (+)	2.31	0.01 (+)	4.19	1.89[a] (-)	8.98	8.98	0.00	9.21	0.23 (-)
Very large VLDL	1.70[a] (+)	1.74	0.04 (-)	4.51	2.81[a] (-)	9.63	9.70	0.07 (-)	10.12	0.49 (-)
Large VLDL	1.54 (+)	1.60	0.06 (-)	5.18	3.64[a] (-)	11.42	11.54	0.12 (-)	12.07	0.65 (-)
Medium VLDL	4.29[a] (+)	4.34	0.05 (+)	7.76	3.47[a] (-)	17.12	17.14	0.02 (+)	17.47	0.35 (-)
Small VLDL	9.79[a] (+)	9.96	0.17 (+)	12.79	3.00[a] (-)	24.08	24.17	0.09 (+)	24.21	0.13 (-)
Very small VLDL	17.68[a] (+)	18.24	0.56 (+)	18.65	0.97 (-)	27.65	28.03	0.38 (+)	27.75	0.10 (+)
IDL	13.12[a] (+)	13.45	0.33 (+)	13.60	0.48 (-)	19.15	19.35	0.20 (+)	19.26	0.11 (+)
Large LDL	12.64[a] (+)	13.00	0.36 (+)	13.15	0.51 (-)	18.74	18.96	0.22 (+)	18.84	0.10 (+)
Medium LDL	13.25[a] (+)	13.59	0.34 (+)	13.86	0.61 (-)	19.83	20.04	0.21 (+)	19.92	0.09 (+)
Small LDL	13.47[a] (+)	13.77	0.30 (+)	14.06	0.59 (-)	19.94	20.11	0.17 (+)	20.02	0.08 (+)
Very large HDL	0.00 (-)	0.00	0.00 (+)	4.39	4.39[a] (+)	10.54	10.56	0.02 (+)	11.45	0.91 (+)
Large HDL	4.52[a] (-)	4.71	0.19 (-)	8.62	4.10[a] (+)	19.61	19.72	0.11 (-)	19.96	0.35 (+)
Medium HDL	6.06[a] (-)	7.41	1.35 (-)	6.45	0.39 (+)	7.69	8.95	1.26 (-)	7.85	0.16 (-)
Small HDL	0.53 (+)	1.57	1.04 (-)	3.38	2.85[a] (-)	6.82	7.96	1.14 (-)	7.49	0.67 (-)
**Lipoprotein particle size**										
VLDL diameter	0.27 (+)	0.28	0.01 (-)	3.90	3.63[a] (-)	9.01	9.04	0.03 (-)	9.78	0.77 (-)
LDL diameter	1.20 (-)	1.67	0.47 (-)	1.26	0.06 (+)	2.33	2.76	0.43 (-)	2.34	0.01 (-)
HDL diameter	3.16[a] (-)	3.19	0.03 (-)	8.44	5.28[a] (+)	20.65	20.65	0.00 (-)	21.25	0.60 (+)
**Apolipoproteins**										
ApoA1	0.11 (-)	0.29	0.18 (-)	1.05	0.94 (+)	3.05	3.22	0.17 (-)	3.17	0.12 (+)
ApoB	13.89[a] (+)	14.48	0.56 (+)	15.48	1.59[a] (-)	26.21	26.60	0.39 (+)	26.22	0.02 (+)
ApoB / ApoA1 ratio	13.89[a] (+)	14.70	0.81 (+)	16.69	2.80[a] (-)	31.17	31.74	0.57 (+)	31.17	0.00 (-)
**Continues on the next page**										

Fat%, body fat percentage.

+ or – values indicate the direction of association for age and for additional effect of the strength measurements.

[a]Statistically significant association (*P*≤.002) between age or the additional muscular strength variable (independent) and the metabolome measure (dependent) in the model.

© 2019 Kujala UM et al. *JAMA Network Open.*

eTable 14 continues from the previous page

Metabolome measure	R^2 (%) for age	R^2 (%) for age and absolute strength	Additional R^2 (%) for absolute strength after age	R^2 (%) for age and relative strength	Additional R^2 (%) for relative strength after age	R^2 (%) for age and fat%	R^2 (%) for age, fat% and absolute strength	Additional R^2 (%) for absolute strength after age and fat%	R^2 (%) for age, fat% and relative strength	Additional R^2 (%) for relative strength after age and fat%
**Triglycerides**										
Total triglycerides	6.24[a] (+)	6.31	0.07 (+)	9.74	3.50[a] (-)	20.97	20.99	0.02 (+)	21.21	0.24 (-)
VLDL-TG	3.93[a] (+)	3.96	0.03 (+)	7.85	3.92[a] (-)	18.03	18.03	0.00 (+)	18.44	0.41 (-)
IDL-TG	14.24[a] (+)	14.33	0.09 (+)	15.40	1.16 (-)	24.70	24.73	0.03 (+)	24.75	0.05 (+)
LDL-TG	18.63[a] (+)	18.80	0.17 (+)	19.48	0.85 (-)	27.53	27.62	0.09 (+)	27.62	0.09 (+)
HDL-TG	6.86[a] (+)	7.02	0.16 (+)	7.86	1.00 (-)	12.88	13.00	0.12 (+)	12.90	0.02 (-)
**Cholesterol**										
Total cholesterol	10.68[a] (+)	10.92	0.24 (+)	10.78	0.10 (-)	14.02	14.16	0.14 (+)	14.16	0.14 (+)
VLDL-C	11.93[a] (+)	12.32	0.39 (+)	13.88	1.95[a] (-)	23.72	23.99	0.27 (+)	23.74	0.02 (-)
IDL-C	11.44[a] (+)	11.82	0.38 (+)	11.80	0.36 (-)	16.65	16.88	0.23 (+)	16.77	0.12 (+)
LDL-C	11.82[a] (+)	12.16	0.34 (+)	12.24	0.42 (-)	17.11	17.32	0.21 (+)	17.21	0.10 (+)
HDL-C	2.20[a] (-)	2.66	0.46 (-)	4.66	2.46[a] (+)	11.83	12.19	0.36 (-)	11.99	0.16 (+)
HDL_2-C	3.63[a] (-)	4.05	0.42 (-)	6.34	2.71[a] (+)	14.47	14.79	0.32 (-)	14.63	0.16 (+)
HDL_3-C	6.59[a] (+)	6.75	0.16 (-)	6.64	0.05 (+)	6.53	6.70	0.17 (-)	6.65	0.12 (+)
Esterified cholesterol	10.20[a] (+)	10.40	0.20 (+)	10.30	0.10 (-)	13.41	13.52	0.11 (+)	13.53	0.12 (+)
Free cholesterol	11.79[a] (+)	12.14	0.35 (+)	11.89	0.10 (-)	15.40	15.63	0.23 (+)	15.59	0.19 (+)
**Fatty acids**										
Total fatty acids	12.43[a] (+)	12.55	0.12 (+)	13.59	1.16 (-)	21.53	21.59	0.06 (+)	21.53	0.00 (+)
Unsaturation degree	1.91[a] (-)	2.70	0.79 (+)	7.26	5.35[a] (+)	12.05	12.96	0.91 (+)	13.50	0.45 (+)
Omega-3 FA	13.81[a] (+)	15.64	1.83[a] (+)	13.76	-0.00	17.08	18.66	1.58[a] (+)	17.83	0.75 (+)
Omega-3 FA ratio	4.97[a] (+)	8.64	3.67[a] (+)	7.35	2.38[a] (+)	5.27	8.83	3.56[a] (+)	7.36	2.09[a] (+)
Docosahexaenoic acid	13.12[a] (+)	14.59	1.47[a] (+)	13.05	0.00	15.79	17.02	1.23 (+)	16.55	0.76 (+)
Polyunsaturated FA	12.19[a] (+)	12.54	0.35 (+)	12.33	0.14 (-)	16.53	16.76	0.23 (+)	16.68	0.15 (+)
Omega-6 FA	11.42[a] (+)	11.62	0.20 (+)	11.64	0.22 (-)	15.78	15.89	0.11 (+)	15.87	0.09 (+)
Omega-6 FA ratio	3.54[a] (-)	3.58	0.04 (-)	6.61	3.07[a] (+)	16.99	17.01	0.02 (-)	17.17	0.18 (+)
Linoleic acid	10.13[a] (+)	10.20	0.07 (+)	10.42	0.29 (-)	13.95	13.97	0.02 (+)	13.98	0.03 (+)
Saturated FA	12.16[a] (+)	12.35	0.19 (+)	13.27	0.11 (-)	21.39	21.49	0.10 (+)	21.39	0.00 (+)
Saturated FA ratio	0.10 (+)	0.42	0.32 (+)	0.08	0.00	0.45	0.76	0.31 (+)	0.51	0.06 (+)
Monounsaturated FA	9.79[a] (+)	9.80	0.01 (+)	12.42	2.63[a] (-)	22.90	22.90	0.00 (-)	23.00	0.10 (-)
**Continues on the next page**										

Fat%, body fat percentage.
+ or – values indicate the direction of association for age and for additional effect of the strength measurements.
[a]Statistically significant association (*P*≤.002) between age or the additional muscular strength variable (independent) and the metabolome measure (dependent) in the model.

© 2019 Kujala UM et al. *JAMA Network Open.*

eTable 14 continues from the previous page

Metabolome measure	R^2 (%) for age	R^2 (%) for age and absolute strength	Additional R^2 (%) for absolute strength after age	R^2 (%) for age and relative strength	Additional R^2 (%) for relative strength after age	R^2 (%) for age and fat%	R^2 (%) for age, fat% and absolute strength	Additional R^2 (%) for absolute strength after age and fat%	R^2 (%) for age, fat% and relative strength	Additional R^2 (%) for relative strength after age and fat%
**Metabolic substrates**										
Glucose	5.05[a] (+)	5.85	0.80 (+)	4.97	0.00	6.16	6.85	0.69 (+)	6.32	0.16 (+)
Glycerol	0.35 (+)	1.77	1.42 (-)	7.85	7.50[a] (-)	19.73	21.47	1.74[a] (-)	21.16	1.43[a] (-)
Acetoacetate	0.17 (-)	3.55	3.38[a] (-)	2.17	2.00[a] (-)	0.39	3.93	3.54[a] (-)	2.18	1.79[a] (-)
3-hydroxybuturate	0.12 (-)	3.41	3.29[a] (-)	2.17	2.05[a] (-)	1.29	4.82	3.53[a] (-)	2.53	1.24 (-)
Acetate	0.29 (+)	0.29	0.00 (-)	0.29	0.00	0.30	0.30	0.00 (-)	0.30	0.00 (-)
Citrate	0.06 (+)	0.72	0.66 (-)	1.02	0.94 (-)	0.66	1.41	0.75 (-)	1.21	0.55 (-)
Lactate	0.79 (+)	0.89	0.10 (-)	2.79	2.00 (-)	5.68	5.83	0.15 (-)	6.08	0.40 (-)
Pyruvate	0.00 (-)	0.87	0.87 (-)	3.39	3.39[a] (-)	4.24	5.37	1.13 (-)	5.59	1.35 (-)
**Amino acids**										
Isoleucine (BCAA)	0.00 (-)	0.45	0.45 (+)	2.10	2.10[a] (-)	12.10	12.45	0.35 (+)	12.13	0.03 (-)
Leucine (BCAA)	0.31 (-)	0.97	0.66 (+)	2.08	1.77[a] (-)	13.40	13.95	0.55 (+)	13.40	0.00 (+)
Valine (BCAA)	2.05[a] (-)	3.23	1.18 (+)	2.39	0.34 (-)	7.72	8.82	0.10 (+)	7.90	0.18 (+)
Alanine	1.56 (+)	2.27	0.71 (+)	2.28	0.72 (-)	7.44	8.03	0.59 (+)	7.45	0.01 (+)
Glutamine	17.36[a] (-)	17.97	0.61 (-)	17.65	0.29 (-)	22.09	22.56	0.47 (-)	22.20	0.11 (-)
Glycine	0.06 (-)	8.64	8.58 (-)	0.06	0.00	2.72	3.38	0.66 (-)	2.98	0.26 (-)
Histidine	0.74 (-)	0.78	0.04 (+)	1.08	0.34 (-)	1.57	1.60	0.03 (+)	1.60	0.03 (-)
Phenylalanine	0.02 (+)	1.82	1.80[a] (+)	1.34	1.32 (-)	18.87	20.59	1.72[a] (+)	19.11	0.24 (+)
Tyrosine	0.12 (+)	4.95	4.83[a] (+)	0.17	0.05 (-)	8.98	13.72	4.74[a] (+)	9.94	0.96 (+)
**Miscellaneous**										
Glycoproteins	0.16 (+)	0.17	0.01 (-)	6.02	5.86[a] (-)	22.58	22.60	0.02 (-)	23.09	0.51 (-)
Creatinine	1.02 (+)	6.76	5.74[a] (+)	4.97	3.95[a] (+)	1.18	7.08	5.90[a] (+)	5.06	3.88[a] (+)
Albumin	20.03[a] (-)	20.52	0.49 (-)	20.59	0.56 (-)	20.49	20.99	0.50 (-)	20.68	0.19 (-)

Fat%, body fat percentage.
+ or – values indicate the direction of association for age and for additional effect of the strength measurements.
[a]Statistically significant association ($P \leq .002$) between age or the additional muscular strength variable (independent) and the metabolome measure (dependent) in the model.

© 2019 Kujala UM et al. *JAMA Network Open.*

eTable 15. Regression Models Including Age, Body Fat Percentage, Aerobic Fitness, and Relative Muscular Strength

Metabolome measure	Association of aerobic fitness adjusted for age, fat%, and relative muscular strength		Association of relative muscular strength adjusted for age, fat%, and aerobic fitness	
	β[a]	*P*[a]	β[a]	*P*[a]
**Lipoprotein particle concentration**				
Extremely large VLDL	-0.02702	2.310e-02	-0.03752	0.5724
Very large VLDL	-0.03517	8.151e-02	-0.1393	0.2178
Large VLDL	-0.02082	2.336e-02	-0.07193	0.1614
Medium VLDL	-0.009731	2.877e-03	-0.01305	0.4736
Small VLDL	-0.005574	5.994e-03	-0.002543	0.8223
Very small VLDL	-7.421e-11	0.1092	3.279e-10	0.2061
IDL	-9.446e-11	0.5102	8.182e-10	0.3085
Large LDL	-1.508e-10	0.5553	1.384e-09	0.3340
Medium LDL	-1.439e-10	0.5162	1.117e-09	0.3681
Small LDL	-1.366e-10	0.5791	1.200e-09	0.3850
Very large HDL	3.222e-09	3.646e-04[b]	7.210e-09	0.1525
Large HDL	1.145e-08	1.493e-07[b]	2.008e-09	0.8678
Medium HDL	6.552e-09	1.456e-04[b]	-1.866e-08	5.225e-02
Small HDL	7.241e-10	0.7717	-2.850e-08	4.176e-02
**Lipoprotein particle size**				
VLDL diameter	-0.02262	5.798e-03	-0.06451	0.1591
LDL diameter	5.102e-04	0.6151	-0.002153	0.7047
HDL diameter	0.006227	3.072e-06[b]	0.006127	0.4080
**Apolipoproteins**				
ApoA1	0.003876	1.485e-05[b]	-0.001649	0.7402
ApoB	-0.002223	3.183e-02	0.004656	0.4212
ApoB / ApoA1 ratio	-0.003359	2.069e-05[b]	0.004053	0.3553
**Triglycerides**				
Total triglycerides	-0.007227	2.296e-03	-0.006388	0.6290
VLDL-TG	-0.009790	1.677e-03	-0.01435	0.4090
IDL-TG	-0.002713	5.326e-02	0.008703	0.2677
LDL-TG	-2.293e-04	0.2683	0.001283	0.2688
HDL-TG	-0.001185	0.4111	-0.0007832	0.9227
**Cholesterol**				
Total cholesterol	0.002527	0.6064	0.02169	0.4298
VLDL-C	-0.005008	2.469e-02	0.002860	0.8184
IDL-C	-5.492e-04	0.5904	0.005705	0.3182
LDL-C	-0.001224	0.6597	0.01414	0.3640
HDL-C	0.007489	9.175e-07[b]	-0.002278	0.7876
HDL_2-C	0.006958	6.949e-07[b]	-0.002191	0.7779
HDL_3-C	.4.793e-04	8.628e-03	0.0001488	0.8839
Esterified cholesterol	0.001996	0.5736	0.01416	0.4760
Free cholesterol	5.282e-04	0.6997	0.007553	0.3249
**Continues on the next page**				

Fat%, body fat percentage.
[a]Unstandardised regression coefficients (β) and *P* values for aerobic fitness and relative muscular strength in the model adjusted for age, fat percentage, and each other (independent variables) in relation to each metabolome measure (dependent variable). In the documentation of regression coefficient note that 7.421e-11 means 0.00000000007421 and in the documentation of *P* values note that *P*=2.310e-02 means *P*=0.02310 etc.
[b]*P* value is statistically significant at the level of *P*≤.002.

© 2019 Kujala UM et al. *JAMA Network Open.*

eTable 15 continues from the previous page

Metabolome measure	Association of aerobic fitness adjusted for age, fat%, and relative muscular strength		Association of relative muscular strength adjusted for age, fat%, and aerobic fitness	
	β[a]	*P*[a]	β[a]	*P*[a]
**Fatty acids**				
Total fatty acids	-6.396e-04	0.5950	0.001417	0.8335
Unsaturation degree	0.001045	5.286e-04[b]	0.003519	3.666e-02
Omega-3 FA	6.385e-04	0.2955	0.006565	5.510e-02
Omega-3 FA ratio	0.01068	5.667e-03	0.05911	6.262e-03
Docosahexaenoic acid	1.586e-04	0.4165	0.002162	4.831e-02
Polyunsaturated FA	0.002494	0.5257	0.01793	0.4154
Omega-6 FA	0.001854	0.5858	0.01136	0.5512
Omega-6 FA ratio	0.04654	3.344e-03	0.02836	0.7487
Linoleic acid	0.002022	0.5016	0.004797	0.7759
Saturated FA	-0.001132	0.3607	0.002818	0.6846
Saturated FA ratio	-0.01762	4.324e-02	5.307e-02	0.2766
Monounsaturated FA	-0.002099	0.1774	-0.004030	0.6436
**Metabolic substrates**				
Glucose	-0.001220	0.5761	0.01317	0.2814
Glycerol	-0.007666	4.014e-05[b]	-0.02151	3.897e-02
Acetoacetate	-0.01717	6.775e-05[b]	-0.05014	3.674e-02
3-hydroxybuturate	-0.01183	8.400e-04[b]	-0.03434	8.249e-02
Acetate	5.523e-04	0.7093	-0.001882	0.8205
Citrate	9.562e-05	0.2374	-9.190e-04	4.283e-02
Lactate	0.002383	0.1711	-0.01812	6.323e-02
Pyruvate	-0.001827	0.1673	-0.01746	1.796e-02
**Amino acids**				
Isoleucine (BCAA)	-0.001198	0.3775	-0.001406	0.8532
Leucine (BCAA)	-7.800e-04	0.4414	0.001531	0.7872
Valine (BCAA)	6.128e-05	0.7003	8.211e-04	0.3572
Alanine	3.907e-04	0.2013	-0.0002611	0.8787
Glutamine	0.001098	1.610e-02	-0.003881	0.1261
Glycine	6.564e-04	0.4135	-6.271e-03	0.1633
Histidine	2.455e-05	0.5732	-1.387e-04	0.5699
Phenylalanine	3.447e-04	0.6217	0.004465	0.2539
Tyrosine	2.403e-05	0.6695	7.096e-04	2.471e-02
**Miscellaneous**				
Glycoproteins	-0.003280	2.900e-05[b]	-0.003490	0.4237
Creatinine	1.315e-06	0.9814	1.4676e-03	4.304e-06[b]
Albumin	3.255e-05	0.2436	-2.241e-04	0.1518

Fat%, body fat percentage.
[a]Unstandardised regression coefficients (β) and *P* values for aerobic fitness and relative muscular strength in the model adjusted for age, fat percentage, and each other (independent variables) in relation to each metabolome measure (dependent variable). In the documentation of regression coefficient note that 7.421e-11 means 0.00000000007421 and in the documentation of *P* values note that *P*=2.310e-02 means *P*=0.02310 etc.
[b]*P* value is statistically significant at the level of *P*≤.002.

© 2019 Kujala UM et al. *JAMA Network Open.*
